# CD73 sustained cancer-stem-cell traits by promoting SOX9 expression and stability in hepatocellular carcinoma

**DOI:** 10.1186/s13045-020-0845-z

**Published:** 2020-02-05

**Authors:** Xiao-Lu Ma, Bo Hu, Wei-Guo Tang, Su-Hong Xie, Ning Ren, Lin Guo, Ren-Quan Lu

**Affiliations:** 1 Department of Clinical Laboratory, Fudan University Shanghai Cancer Center, Shanghai, 200032 China; 2 Department of Liver Surgery, Liver Cancer Institute, Zhongshan Hospital, Fudan University, Shanghai, 200032 China; 3 Department of Hepatobiliary and Pancreatic Surgery, Minhang Hospital, Fudan University, Shanghai, 201100 China; 4 Development of Oncology, Shanghai Medical School, Fudan University, Shanghai, 200032 China

**Keywords:** Hepatocellular carcinoma, Cancer stem cells, CD73, AKT signaling, Lenvatinib resistance

## Abstract

**Background:**

Aberrant AKT activation contributes to cancer stem cell (CSC) traits in hepatocellular carcinoma (HCC). We previously reported that CD73 activated AKT signaling via the Rap1/P110β cascade. Here, we further explored the roles of CD73 in regulating CSC characteristics of HCC.

**Methods:**

CD73 expression modulations were conducted by lentiviral transfections. CD73+ fractions were purified by magnetic-based sorting, and fluorescent-activated cell sorting was used to assess differentiation potentials. A sphere-forming assay was performed to evaluate CSC traits in vitro, subcutaneous NOD/SCID mice models were generated to assess in vivo CSC features, and colony formation assays assessed drug resistance capacities. Stemness-associated gene expression was also determined, and underlying mechanisms were investigated by evaluating immunoprecipitation and ubiquitylation.

**Results:**

We found CD73 expression was positively associated with sphere-forming capacity and elevated in HCC spheroids. CD73 knockdown hindered sphere formation, Lenvatinib resistance, and stemness-associated gene expression, while CD73 overexpression achieved the opposite effects. Moreover, CD73 knockdown significantly inhibited the in vivo tumor propagation capacity. Notably, we found that CD73+ cells exhibited substantially stronger CSC traits than their CD73– counterparts. Mechanistically, CD73 exerted its pro-stemness activity through dual AKT-dependent mechanisms: activating SOX9 transcription via c-Myc, and preventing SOX9 degradation by inhibiting glycogen synthase kinase 3β. Clinically, the combined analysis of CD73 and SOX9 achieved a more accurate prediction of prognosis.

**Conclusions:**

Collectively, CD73 plays a critical role in sustaining CSCs traits by upregulating SOX9 expression and enhancing its protein stability. Targeting CD73 might be a promising strategy to eradicate CSCs and reverse Lenvatinib resistance in HCC.

## Introduction

Hepatocellular carcinoma (HCC) ranks as the leading lethal malignant tumor worldwide [1–3]. Though great improvements have been made recently, the prognosis of HCC patients remains unfavorable, as evidenced by 25–30% overall 5-year survival and 50–70% recurrence/metastasis rates within 5 years after radical resection [4–7]. However, the detailed mechanisms underlying liver cancer carcinogenesis and progression remain largely unknown. Hence, the identification of key mediators of HCC development and progression, and the clarification of molecular mechanisms could help improve HCC patient prognosis.

Accumulating evidence has revealed that cellular heterogeneity is maintained in most solid tumors including HCC [8–12]. Cancer stem cells (CSCs) are a small proportion of tumor cells that exhibit capacities of self-renewal and differentiation [13, 14]. In HCC, CSCs initiate tumor development, induce tumor progression and modulate chemotherapy resistance [15–17]. Therefore, targeting therapy to eradicate CSCs has the potential to hinder HCC progression. Aberrant signaling activation is involved in maintaining CSC traits in distinct cancers such as gastric and colorectal carcinoma, and HCC [18]. The activation of AKT signaling is implicated in HCC initiation and considered a hallmark to reflect the acquisition of CSC traits [19–22]. However, it is unclear how key upstream regulators of AKT signaling confer CSC traits to HCC cells.

We previously identified CD73 as a novel indicator of poor prognosis in HCC [23]. Importantly, CD73 promoted HCC progression and metastasis by activating AKT signaling [23], leading us to hypothesize that CD73 could serve as a novel biomarker for CSCs in HCC. Here, we report that CD73 expression is essential for maintaining CSC traits in HCC cells and that CD73+ HCC cells exhibit substantially greater CSC potential than their CD73– counterparts. Further investigation demonstrated that CD73 not only promotes the transcription of SOX9, but also increases its protein stability in an AKT/glycogen synthase kinase (GSK)3β-dependent manner, which sustained the stemness of HCC cells.

## Materials and methods

### Patients and clinical samples

Primary HCC samples were obtained from cohort 1 (*n* = 25, collected from April to June 2018; fresh cancerous tissues were collected and used for sphere-forming assays); cohort 2 (*n* = 7, collected from July to August 2018; fresh cancerous tissues were collected and used for CD73+ sorting); cohort 3 (*n* = 212, collected from January to December 2011; used for immunohistochemistry [IHC] staining). All enrolled patients had undergone curative resection at Zhongshan Hospital.

The present study was approved by the Zhongshan Hospital Research Ethics Committee, and all individuals provided their informed consent. HCC diagnosis was based on histopathology examination according to the American Association for Study of Liver Disease guidelines. Follow-up was conducted as previously described [24] and ended in December 2018. Time to recurrence (TTR) and overall survival (OS) were defined according to previous reports [23–25].

### Cell lines and animals

HCC cell lines HCCLM3, Hep3B, MHCC97L, and HepG2 were from Yang Xu (Zhongshan Hospital, Fudan University). All cell lines were cultured in DMEM medium supplemented with 10% FBS, 100 unit/ml streptomycin, 100 μg/ml penicillin. Male 4- to 6-week-old non-obese diabetic severe combined immunodeficiency (NOD-SCID) mice were obtained from the Chinese Academy of Science. All model mice were maintained in specific pathogen-free conditions. Humane care of animals was objected to the “Guide for the Care and Use of Laboratory Animals” criteria of the National Academy of Science (National Institute of Health publication 86-23, revised 1985) [26].

### Preparation of primary HCC cells

Primary single HCC cell suspensions were obtained according to our previous study [27].

### Sphere-forming assays

Sphere-forming assays were conducted according to our previous study [27]. For HCC cell line culture, cells were seeded at a density of 2000 per well in a 6-well plate. For primary HCC cell culture, tumor cells were seeded at a density of 20,000 per well in a 6-well plate.

### RT-PCR and western blot assays

Total RNA extraction was performed using a RNeasy mini kit (Qiagen, Germany), while cDNA synthesis was performed using the Quantitect Reverse Transcription Kit (Qiagen, Germany) as we did previously [23, 27]. mRNA expressions of Target genes as well as internal control were determined with FastStart Universal SYBR Green Master (Roche diagnostic, Germany) and Lightcycle 480 (Roche diagnostic, Germany). ΔCq method (Cq^target^–Cq^contorl^) was applied to quantify the relative expression of target genes. Primers used in the present study were listed in Additional file 1: Table S1.

western blot (WB) assays were performed according to our previous studies [23, 28]. Antibodies and corresponding dilutions were listed in Additional file 1: Table S2. All experiments were conducted in triplicate.

### Transfections

The hU6-MCS-CMV-Puromycin lentiviral vectors were purchased from GeneChem Co Ltd. (Shanghai, China). Two distinct shRNAs targeting CD73 and three shRNAs targeting Sox9 were purchased from Merdiobio Co Ltd. (Shanghai, China). shRNA for c-Myc was purchased from Santa Cruz (USA). shRNAs were cloned into lentiviral vectors according to the manufacturer’s instructions. Lentivirus were further transfected into indicated HCC cell lines, and the knockdown efficiencies were validated by WB assays. Both two shRNAs targeting CD73 exerted satisfactory effects, which was in accordance with our previous study [23]. #1 and #2 shRNAs targeting Sox9 were selected for further experiments due to their satisfactory knockdown efficiencies.

For overexpression, expression plasmid of CD73, wild-type SOX9, and mutant SOX9 (T236A) were obtained from Merdiobio Co Ltd. (Shanghai, China). Stable overexpression was verified by WB assays.

### Evaluations of self-renewal and differentiated capacities

Differentiated capacity was assessed via culturing sphere cells in medium supplemented with 10% FBS according to our previous study and expression of stemness-associated genes were determined by RT-PCR [27]. EpCAM was set as an internal control reflecting CSC traits due to its universal expression pattern in HCC stem cells [29]. For self-renewal capacity evaluation, serial sphere formation was conducted in continuous 6 passages according to previous studies [27, 30].

### In vivo serial dilution xenograft tumor formation

NOD-SCID mice were randomly divided into groups (six per group) and maintained in SPF environment [27]. After quantification of cell number, indicated HCC cells were suspended in a DMEM/MatriGel (Corning, USA) mixture (volume ratio, 1:1), and then injected subcutaneously into the flanks of recipient mice. Tumor formation was monitored as previously described [26, 27], and the incidence was recorded. In vivo tumorigenicity assessment was terminated 12 weeks after injection, at which point mice with no obvious tumor nodules observed at the injection site were considered as negative results.

### Isolation of CD73+ cells by magnetic bead cell sorting

For magnetic cell sorting, HCC cells were labeled with primary CD73 antibody (Abcam, USA) followed by incubation with anti-mouse IgG microbeads according to the manufacturer’s instructions. Then, MACS was carried out with miniMACS Starting Kit according to the operation handbook of the manufacturer. To achieve a high purity (> 95%), we conducted positive selection with LS column three times. Aliquots of CD73+ and CD73− populations were evaluated for purity with a FACS Aria II (BD Biosciences). For CD73+ proportion assessment, indicated cells were also labeled with primary CD73 antibody followed by incubation with FITC-conjugated anti-mouse IgG (Abcam, USA). Then FACS analysis was performed with a FACS Aria II. All experiments were conducted in triplicate.

### Colony formation assays

For Lenvatinib resistance evaluations, colony formation assays were performed according to our previous studies with tiny modification [23]. Indicated cells were seeded at a density of 4000 cells per well in a 6-well plate.

### Drugs and reagents

Lenvatinib was purchased from Selleck and applied as indicated concentrations. Giemsa staining solution was obtained from Sigma. MK-2206 (10 μM) and SC-79 (5 μM) were purchased from Selleck. 10058-F4 (5μM) was purchased from MCE.

### Luciferase reporter assays

The luciferase reporter assays were performed as the previous study did [31]. The luciferase activities were assessed with Dual-Luciferase Reporter Assay System (Promega, USA) according to the manufacturer’s instructions at 48 h after transfection. All experiments were performed in triplicate.

### Immunohistochemistry staining

Immunohistochemistry IHC staining was conducted with a tissue microarray according to our previous study [23]. The detailed dilution ratio of antibodies used could be seen in Additional file 1: Table S2. Negative controls were included in all assays by omitting the primary antibody. Quantification criteria of IHC staining were the same as the previous study by two independent pathologists [28].

### TCGA database mining

mRNA expression levels of CD73, SOX9, and c-Myc from TCGA database were obtained via StarBase 3.0, and correlations among these three genes were also evaluated based on StarBase 3.0 database [32].

### Immunoprecipitation

Protein complexes were precipitated from whole-cell lysates according to our previous study with specific SOX9 antibody, followed by precipitation with protein G beads (Thermo, USA) [23]. To obtain the immunoprecipitates, beads were further boiled in loading buffer followed by centrifugation to obtain supernatants. To assess the expression levels of certain protein within immuoprecipitates, SDS-PAGE and WB assays were performed as previously report described [23].

### Protein stability with cycloheximide

SOX9 protein stability in different modulated HCC cells was assessed with the treatment of cycloheximide (CHX) (10 μg/ml) for 1, 2, and 8 h as the previous study did [33].

### Ubiquitylation assay

For in vivo ubiquitylation assay, indicated plasmids were transfected into cells along with MG132 treatment (20 μM) for 6 h prior to harvest. Subsequently, protein from whole-cell lysates was extracted followed by sonicated and diluted 10 times with NP-40 lysis buffer according to a previous study [34]. Then, immunoprecipitation was conducted with SOX9 antibody followed by WB assays with indicated antibodies.

### Statistical analysis

Statistical analyses were performed using SPSS 21.0 software (IBM, Chicago, IL, USA). Experimental values for continuous variables were expressed as the mean ± standard error of the mean. The chi-squared test, Fisher’s exact probability tests, and the Student’s *t* test were used as appropriate to evaluate the significance of differences in data between groups. If variances within groups were not homogeneous, a non-parametric Mann–Whitney test was used. Prognostic value was evaluated by Kaplan–Meier survival curves, log-rank tests, and Cox proportional hazards models. A *P* value less than 0.05 was considered significant (Additional file 2).

## Results

### CD73 expression was associated with sphere-forming capacity and was elevated in HCC spheroids

**Fig. 1:**
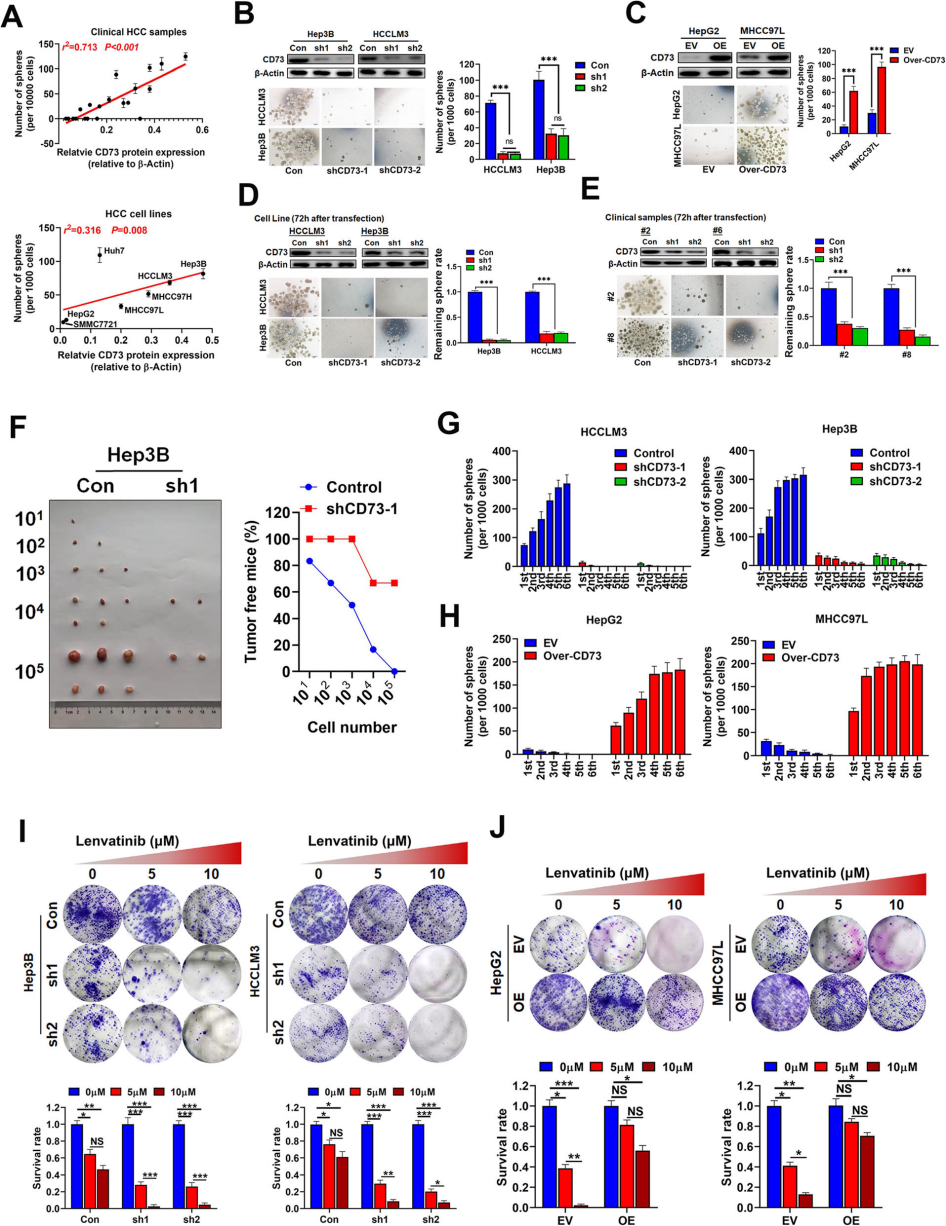
CD73 expression is essential for sustaining CSC traits. **a** Correlations between CD73 expression and number of spheres derived from clinical fresh HCC samples (upper) or HCC cell lines (lower). **b** Effects of CD73 knockdown on sphere-forming capacity in CD73-high HCC cell lines. CD73 knockdown efficiencies were validated by WB assays. **c** Effects of CD73 overexpression on sphere-forming capacity in CD73-low HCC cell lines. CD73 knockdown efficiencies were validated by WB assays. **d** CD73 knockdown interfered sphere-forming capacity in sphere cells derived from HCC cell lines. CD73 knockdown efficiencies were validated by WB assays. **e** CD73 knockdown interfered sphere-forming capacity in sphere cells derived from clinical fresh HCC samples. CD73 knockdown efficiencies were validated by WB assays. **f** Ratio of tumor-free mice after 12 weeks’ tumor formation after injection of indicated numbers of CD73-KD and control Hep3B cells. Images were shown in the left panel. **g** Sphere numbers of CD73-knocked down and control HCC cells according to serial sphere formation assays. **h** Sphere numbers of CD73-overexpressed and control HCC cells according to serial sphere formation assays. **i** Effects of CD73 knockdown on Lenvatinib resistance were evaluated by colony formation assays. Typical images were shown as upper panels. **j** Effects of CD73 overexpression on Lenvatinib resistance were evaluated by colony formation assays. Typical images were shown as upper panels. Throughout the figure, “*” indicated *P* < 0.05, “**” indicated *P* < 0.01, and “***” indicated *P* < 0.001 by two-tailed *t* test or Mann–Whitney test

We first evaluated the association between CD73 expression and sphere-forming capacity in 25 fresh resection HCC samples, of which 12 formed spheres within 2 weeks. CD73 protein expression levels were significantly positively associated with the number of spheres formed (*R*^2^ = 0.736, *P* < 0.001, Fig. 1a upper). Similarly, we detected a significant positive correlation between CD73 protein expression and sphere numbers in six HCC cell lines (*R*^2^ = 0.316, *P* = 0.008, Fig. 1a lower). Moreover, HCC spheroid cells derived from both clinical samples and cell lines exhibited higher CD73 protein expression compared with their parental cells respectively according to western blotting assays (Additional file 3: Figure S1A and B).

### CD73 expression conferred CSC traits to HCC cells

We knocked down CD73 expression in two CD73-high expression HCC cell lines, Hep3B, and HCCLM3, and overexpressed CD73 in two CD73-low expression cell lines, HepG2, and MHCC97L. After carrying out sphere-forming assays, we found that CD73 knockdown greatly hindered sphere formation (Fig. 1b), whereas CD73 overexpression remarkably increased sphere numbers (Fig. 1c). To validate these results, Hep3B and HCCLM3 spheres were transfected with CD73 short hairpin (sh)RNAs. We observed a significant decrease in sphere number 72 h after transfection in both cell lines (Fig. 1d), and similar results were observed in spheres derived from two clinical samples (Fig. 1e). Limiting dilution xenograft assays showed that CD73 knockdown significantly reduced tumor initiation and tumorigenic cell frequency compared with control cells (Fig. 1f).

Serial sphere formation assays revealed that CD73 knockdown also greatly reduced the ability of cells to self-renew (Fig. 1g), whereas CD73 overexpression achieved the opposite effect (Fig. 1h). Three rounds of serial passaging were performed to investigate dynamic changes in CD73 mRNA expression, and the expression of EpCAM as a universal CSC marker was measured as an internal control to reflect CSC traits [29]. CD73 mRNA expression in Hep3B and HCCLM3 cells was significantly upregulated in sphere cells and showed a notable decrease following 10% FBS-induced differentiation (Additional file 4: Figure S2A). Consistently, similar dynamic change patterns in CD73 mRNA expression were detected in cells derived from two clinical samples (Additional file 4: Figure S2B).

Additionally, CD73 knockdown remarkably sensitized HCC cells to Lenvatinib treatment (Fig. 1i), while CD73 overexpression induced Lenvatinib resistance (Fig. 1j). Collectively, these data suggest that CD73 promoted the self-renewal of HCC cells and in vivo tumor propagation.

### CD73 is essential for the HCC stemness-associated phenotype

**Fig. 2:**
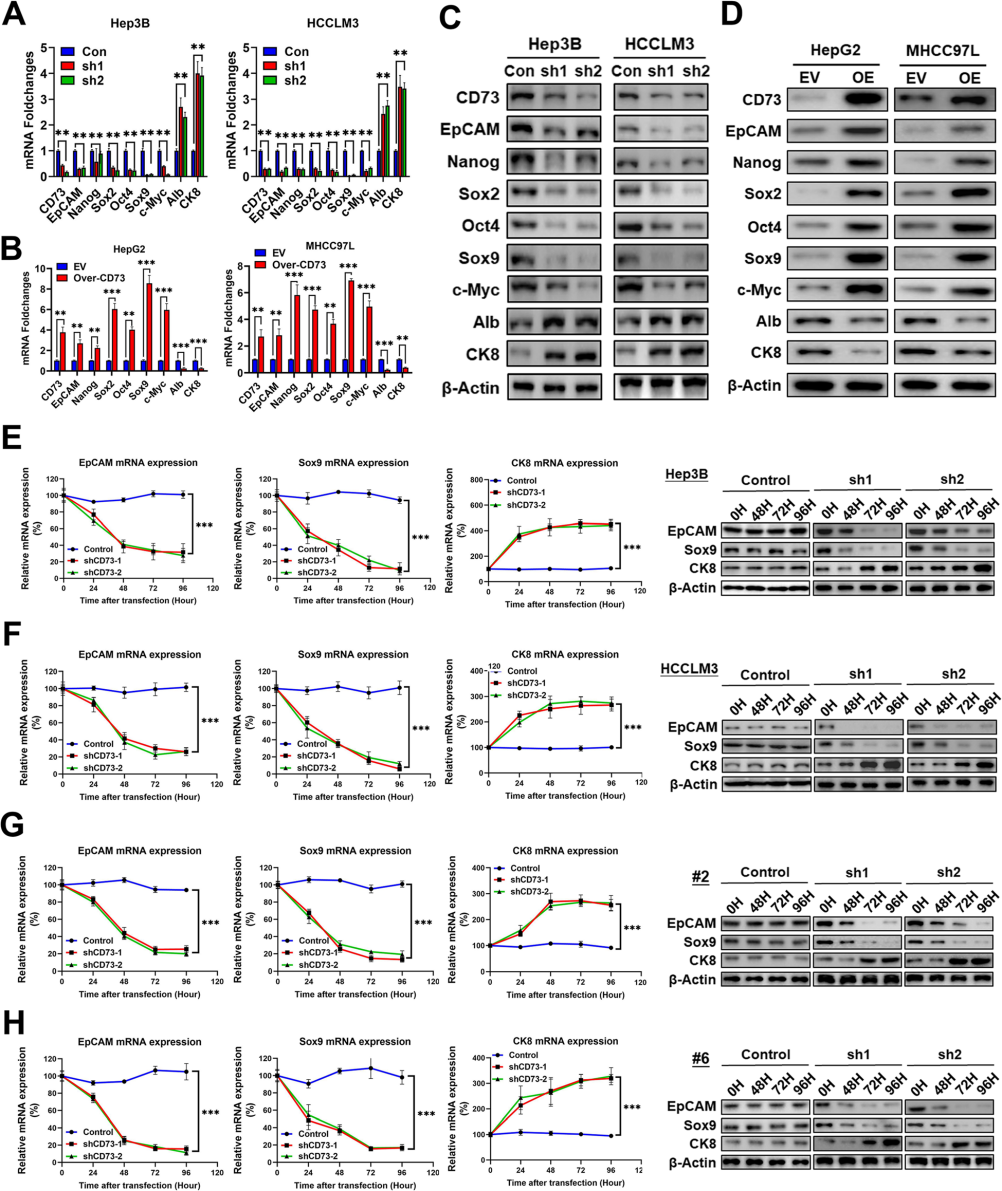
CD73 sustains the stemness-associated molecular phenotype in HCC. **a** Effects of CD73 knockdown on mRNA expressions of stemness-associated genes in Hep3B (left) and HCCLM3 (right) cells were assessed by RT-PCR assays. **b** Effects of CD73 overexpression on mRNA expressions of stemness-associated genes in HepG2 (left) and MHCC97L (right) cells were assessed by RT-PCR assays. **c** Effects of CD73 knockdown on mRNA expressions of stemness-associated genes in Hep3B (left) and HCCLM3 (right) cells were assessed by WB assays. **d** Effects of CD73 overexpression on mRNA expressions of stemness-associated genes in HepG2 (left) and MHCC97L (right) cells were assessed by WB assays. **e** Dynamic changes of EpCAM, SOX9, and CK8 mRNA as well as protein levels after CD73 knockdown in Hep3B spheres were evaluated by RT-PCR and WB assays. **f** Dynamic changes of EpCAM, SOX9, and CK8 mRNA as well as protein levels after CD73 knockdown in HCCLM3 spheres were evaluated by RT-PCR and WB assays. **g** Dynamic changes of EpCAM, SOX9, and CK8 mRNA as well as protein levels after CD73 knockdown in clinical #2 spheres were evaluated by RT-PCR and WB assays. **h** Dynamic changes of EpCAM, SOX9, and CK8 mRNA as well as protein levels after CD73 knockdown in clinical #6 spheres were evaluated by RT-PCR and WB assays. Throughout the figure, “*” indicated *P* < 0.05, “**” indicated *P* < 0.01, and “***” indicated *P* < 0.001 by two-tailed *t* test or Mann–Whitney test

RT-PCR assays indicated that CD73 knockdown significantly reduced the mRNA expression of stemness-associated genes such as EpCAM, Nanog, SOX2, Oct4, SOX9, and c-Myc, while increasing the expression of albumin and cytokeratin 8 (CK8), which were considered mature liver cell markers [27] (Fig. 2a). Conversely, CD73 overexpression achieved the opposite effects (Fig. 2b). Western blotting assays further confirmed these findings (Fig. 2c, d). Notably, after transfection with CD73 shRNAs, the expression of EpCAM and SOX9 decreased in a time-dependent manner in Hep3B and HCCLM3 sphere cells, while CK8 showed increased expression (Fig. 2e, f). Consistently, the transfection of CD73 shRNAs in spheres derived from two clinical HCC samples exerted similar dynamic change patterns of EpCAM, SOX9, and CK8 to cell lines (Fig. 2g, h). These data confirmed that CD73 is essential for maintaining stemness-associated molecular phenotypes in HCC.

### CD73+ HCC cells possess CSC characteristics

**Fig. 3:**
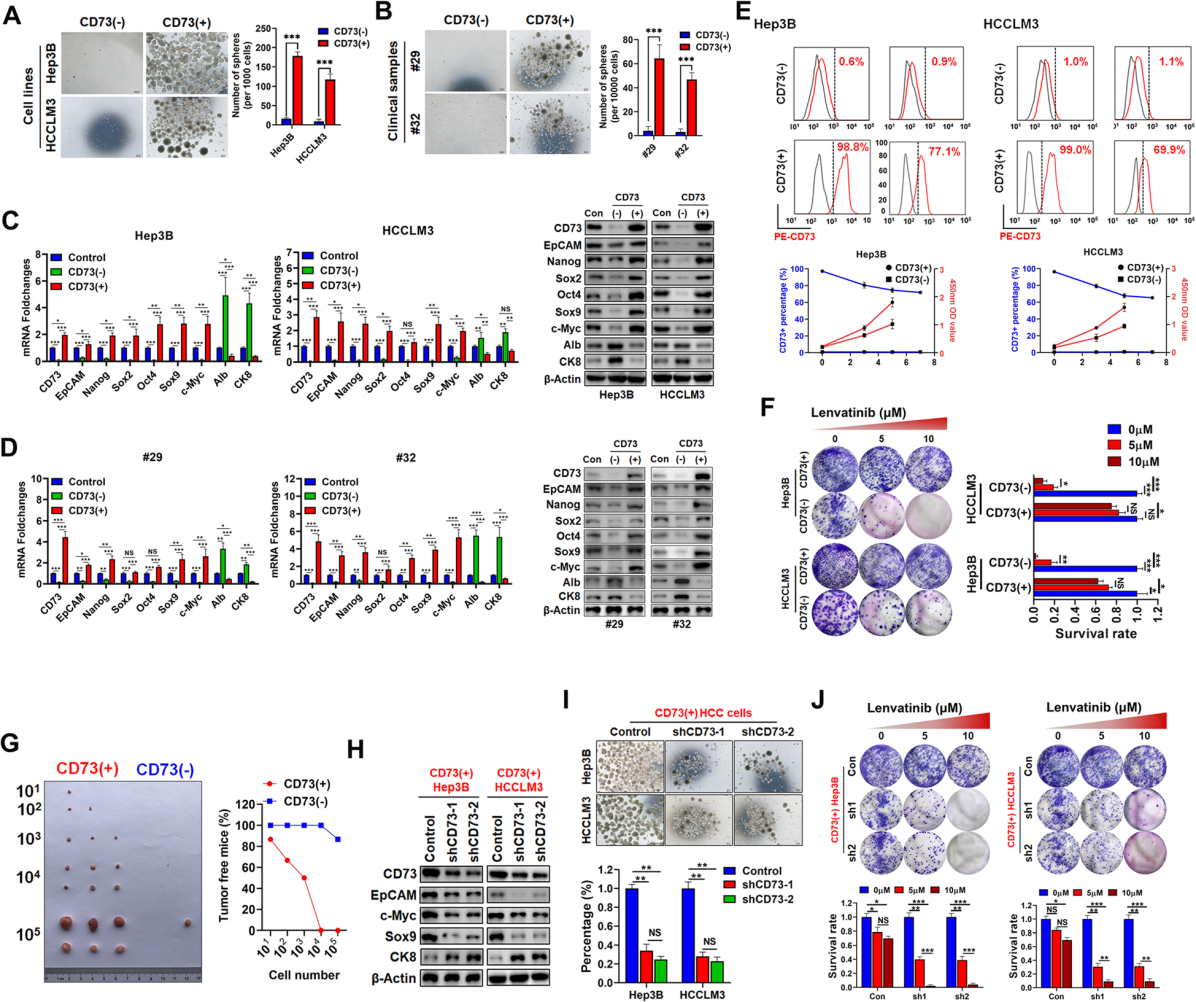
CD73-positive (CD73+) fractions possesses CSC characteristics. **a** Sphere-forming capacities of CD73+ and CD73- fractions purified from HCC cell lines. **b** Sphere-forming capacities of CD73+ and CD73− fractions purified from clinical HCC samples. **c** mRNA and protein expression levels of indicated stemness-associated genes in CD73+ and CD73− fractions purified from HCC cell lines. **d** mRNA and protein expression levels of indicated stemness-associated genes in CD73+ and CD73− fractions purified from clinical HCC samples. **e** Upon culture in serum-containing environment for 7 days, MACS-sorted CD73+ HCC cells gradually reconstitute the original proportion of CD73+ cells, whereas CD73− cells failed to give rise to the original heterogeneity of CD73 expression. **f** Differences of Lenvatinib resistance capacities between CD73+ and CD73− fractions sorted from HCC cell lines were assessed by colony formation assays. Typical images were shown as left panel. **g** Ratio of tumor-free mice after 12 weeks’ tumor formation after injection of indicated numbers of CD73+ and CD73− Hep3B cells. Images were shown in the left panel. **h** Effects of CD73 knockdown on stemness-associated gene expression in CD73+ HCC cells were evaluated by WB assays. **i** Effects of CD73 knockdown on sphere-forming capacities in CD73+ HCC cells. Typical images were shown as upper panel. **j** Effects of CD73 knockdown on Lenvatinib resistance in CD73+ HCC cells were evaluated by colony formation assays. Throughout the figure, “*” indicated *P* < 0.05, “**” indicated *P* < 0.01, and “***” indicated *P* < 0.001 by two-tailed *t* test or Mann–Whitney test

We next examined CD73 expression by FACS in six HCC cell lines. Consistent with western blotting findings, HCCLM3, MHCC97H, and Hep3B cells possessed a high percentage of CD73+ cells, while HepG2, and MHCC97L cells had relatively low CD73+ cell numbers (Additional file 5: Figure S3). MACS-purified CD73– and CD73+ HCC cells underwent sphere formation assays, and CD73+ cells derived from both HCC cell lines and fresh clinical samples were observed to form more spheres than their CD73– counterparts (Fig. 3a, b). Moreover, CD73+ cells exhibited a stemness molecular phenotype, evidenced by the higher expression of stemness-associated genes, while CD73– fractions demonstrated higher expression of albumin and CK8, indicating a mature hepatocyte-like phenotype (Fig. 3c, d). We further purified CD73+ and CD73– cells via MACS, then cultured them in DMEM medium supplemented with 10% FBS for 7 days. CD73 expression was then determined in each subpopulation by FACS. CCK8 assays revealed that both CD73+ and CD73– fractions could proliferate in the given medium. However, CD73+ cells dramatically decreased in number after 3 days, then almost reverted to the presorting level, while CD73– cells maintained their low number after 7 days; this suggested that CD73– cells arose from CD73+ cells and not vice versa (Fig. 3e). CD73+ cells also exhibited greater resistance capacities to Lenvatinib than their CD73– counterparts (Fig. 3f). After purified cells were subcutaneously inoculated into NOD/SCID mice, we observed a difference in tumor incidence between CD73+ and CD73– cells (Fig. 3g).

We further knocked down CD73 expression in purified CD73+ cells, which significantly reduced the expression of stemness-associated genes, while promoting CK8 expression (Fig. 3h). Moreover, CD73 knockdown also greatly hindered the sphere formation capacities of CD73+ cells (Fig. 3i), and induced sensitization to Lenvatnib (Fig. 3j). Taken together, these data suggested that CD73+ cells possess CSC characteristics, and that CD73 expression is essential for sustaining stemness in the CD73+ subpopulation.

### CD73 promotes CSC traits by up-regulating SOX9

**Fig. 4:**
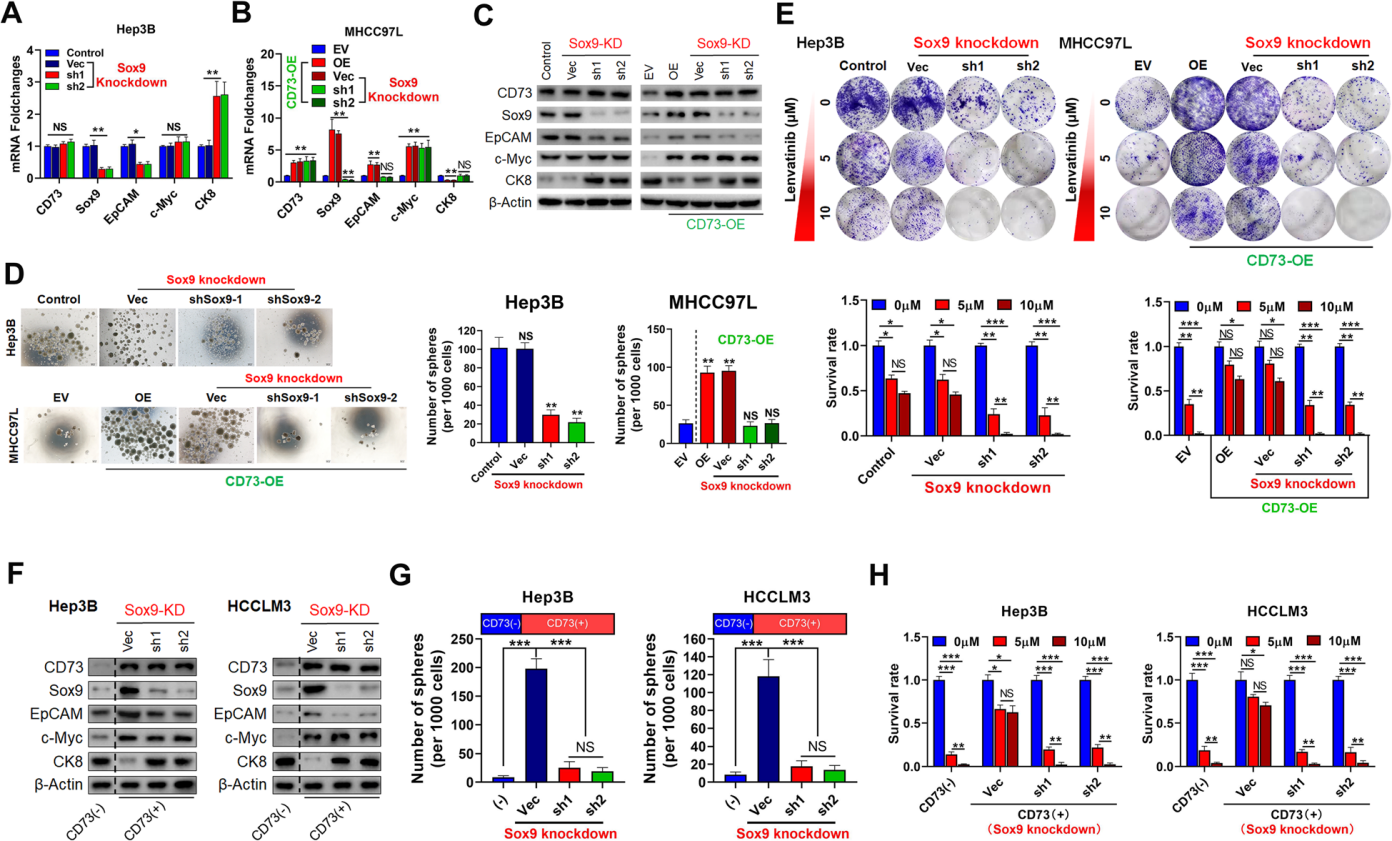
CD73 promotes CSC traits mainly via SOX9. **a** mRNA expressions of indicated genes after SOX9 knockdown in Hep3B cells were evaluated by RT-PCR assays. **b** mRNA expressions of indicated genes after SOX9 knockdown in CD73-overexpressed MHCC97L cells were evaluated by RT-PCR assays. **c** Protein expression levels of indicated genes after SOX9 knockdown in Hep3B (left) and CD73-overexpressed MHCC97L (right) cells were evaluated by WB assays. **d** Effects of SOX9 knockdown on sphere-forming capacities in Hep3B and CD73-overexpressed MHCC97L cells. Typical images of spheres were shown as left panels. (**e**) Effects of SOX9 knockdown on Lenvatinib resistance capacities in Hep3B and CD73-overexpressed MHCC97L cells. Typical images of colony formation assays were shown as upper panels. (**f**) Protein expression levels of stemness-associated genes after SOX9 knockdown in CD73+ cells purified from Hep3B (left) and HCCLM3 (right) cells. **g** Number of spheres after SOX9 knockdown in CD73+ cells purified from Hep3B (left) and HCCLM3 (right) cells. **h** Effects of SOX9 knockdown on Lenvatinib resistance capacities in CD73+ cells purified from Hep3B (left) and HCCLM3 (right) cells. Throughout the figure, “*” indicated *P* < 0.05, “**” indicated *P* < 0.01, and “***” indicated *P* < 0.001 by two-tailed *t* test or Mann–Whitney test

The above data not only revealed that SOX9 mRNA expression was reduced more than that of other stemness-associated genes by CD73 knockdown, but also showed the highest increase after CD73 overexpression in HCC cells (Fig. 2a, b). Interestingly, SOX9 was reported to be regulated by the AKT signaling pathway [35], which is activated by CD73 according to our previous study [23]. Therefore, we speculated that SOX9 could be a key downstream regulator of CD73. To validate this, we first depleted SOX9 expression in CD73-high expression Hep3B cells. EpCAM mRNA expression was significantly decreased, whereas CK8 mRNA expression was greatly upregulated following SOX9 knockdown, which mimicked the effects of CD73 knockdown (Fig. 4a). More importantly, SOX9 knockdown effectively abolished the effects of CD73 on EpCAM and CK8 mRNA expression in MHCC97L cells (Fig. 4b). These findings were further confirmed by western blotting (Fig. 4c). Similarly, SOX9 knockdown mimicked the inhibition effects of CD73 knockdown on sphere formation and Lenvatinib resistance capacities in Hep3B cells and abolished the promotional effects of CD73 overexpression on sphere formation and Lenvatinib resistance capacities in MHCC97L cells (Fig. 4d, e), indicating the potential role of SOX9 in CD73-mediated CSC traits.

We further purified CD73+ cells from two different HCC cell lines, followed by SOX9 shRNA transfection. Western blotting assays indicated that SOX9 knockdown reduced EpCAM expression in CD73+ cells almost to the levels of CD73– cells (Fig. 4f). Further investigation demonstrated that SOX9 knockdown restrained sphere formation and Lenvatinb resistance capacities of CD73+ cells (Fig. 4g, h). Collectively, our data demonstrated that CD73 controlled CSC traits by upregulating SOX9 expression.

### CD73 upregulates SOX9 by AKT signaling

**Fig. 5:**
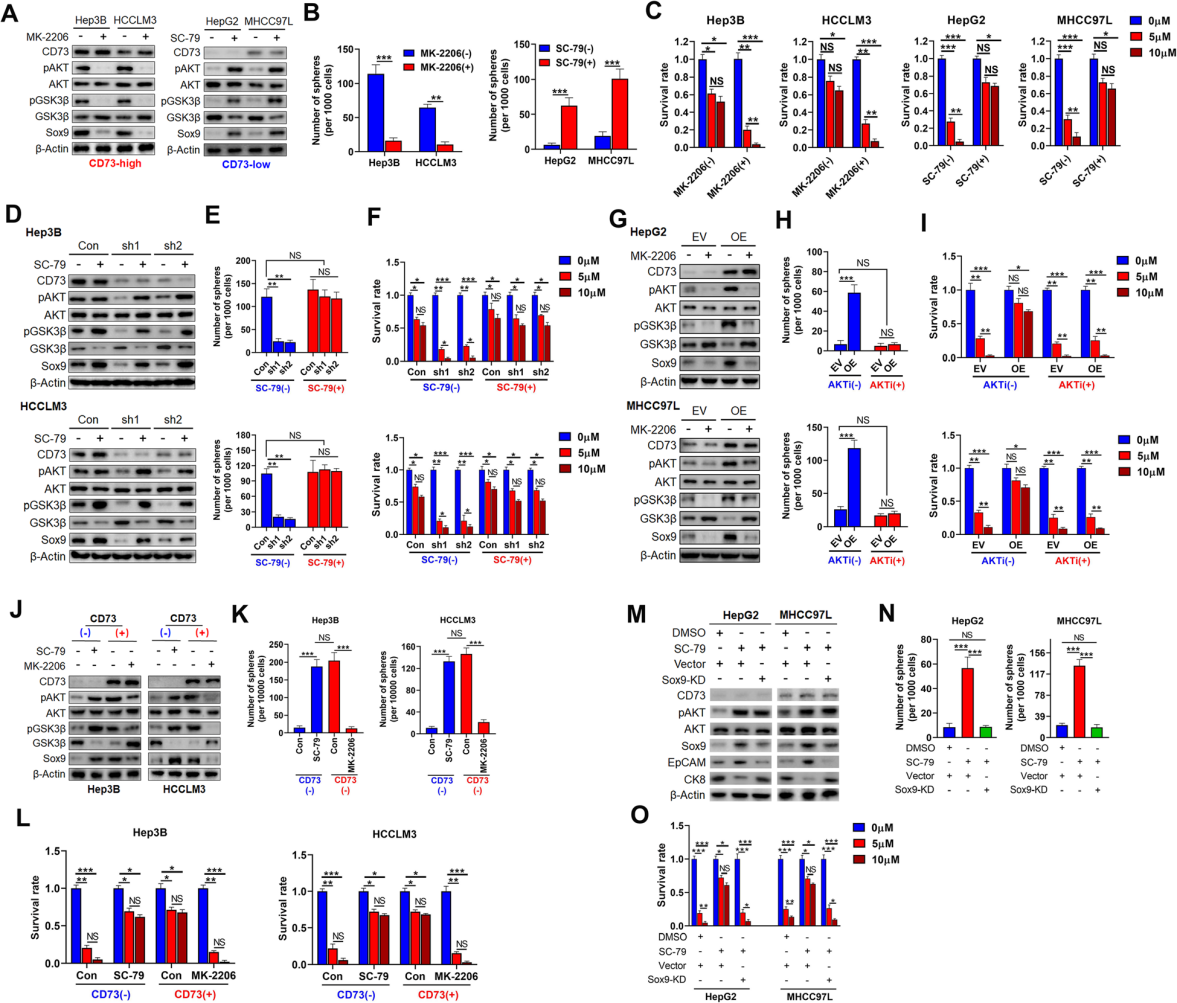
CD73 upregulates SOX9 expression in an AKT-signaling-dependent manner. **a** Effects of AKT antagonist treatment on SOX9 expression in CD73-high HCC cells (left), and effects of AKT agonist treatment on SOX9 expression in CD73-low HCC cells (right). **b** Effects of AKT antagonist treatment on sphere-forming capacities in CD73-high HCC cells (left), and effects of AKT agonist treatment on sphere-forming capacities in CD73-low HCC cells (right). **c** Effects of AKT antagonist treatment on Lenvatinib resistance potentials in CD73-high HCC cells (left two panels), and effects of AKT agonist treatment on Lenvatinib resistance potentials in CD73-low HCC cells (right two panels). **d** Effects of AKT agonist treatment on SOX9 expression in CD73-knockdown HCC cells. **e** Effects of AKT agonist treatment on sphere-forming capacities in CD73-knockdown HCC cells. **f** Effects of AKT agonist treatment on Lenvatinib resistance potentials in CD73-knockdown HCC cells. **g** Effects of AKT antagonist treatment on SOX9 expression in CD73-overexpressed HCC cells. **e** Effects of AKT antagonist treatment on sphere-forming capacities in CD73-overexpressed HCC cells. **f** Effects of AKT antagonist treatment on Lenvatinib resistance potentials in CD73-overexpressed HCC cells. **j** Effects of AKT agonist treatment on SOX9 expression in CD73- HCC cells, and effects of AKT antagonist treatment on SOX9 expression in CD73+ HCC cells. **k** Effects of AKT agonist treatment on sphere-forming capacities in CD73- HCC cells, and effects of AKT antagonist treatment on sphere-forming capacities in CD73+ HCC cells. **l** Effects of AKT agonist treatment on Lenvatinib resistance potentials in CD73- HCC cells, and effects of AKT antagonist treatment on Lenvatinib resistance potentials in CD73+ HCC cells. **m**-**o** Effects of SOX9 knockdown on the expression levels of stemness-associated genes (**m**), sphere-forming capacities (**n**), or Lenvatinib resistance potentials (**o**) in AKT agonist-treated CD73-low HCC cells. Throughout the figure, “*” indicated *P* < 0.05, “**” indicated *P* < 0.01, and “***” indicated *P* < 0.001 by two-tailed *t* test or Mann–Whitney test

We next explored whether CD73 upregulates SOX9 by activating the AKT signaling pathway. First, we treated CD73-high expression cells with the AKT antagonist MK-2206, which greatly reduced pAKT (Ser473) and pGSK3 (Ser9) levels. Importantly, SOX9 expression was also dramatically reduced by AKT inhibition (Fig. 5a). By contrast, treatment with the AKT agonist SC-79 significantly increased SOX9 expression in CD73-low expression cells (Fig. 5a). Additionally, as expected, AKT antagonist treatment significantly restrained sphere formation and Lenvatinib resistance capacities of CD73-high expression cells, whereas the AKT agonist achieved the opposite effects in CD73-low expression cells (Fig. 5b, c). Further study showed that re-activation of AKT by SC-79 successfully rescued SOX9 expression after inhibition by CD73 knockdown, along with restored sphere formation and Lenvatinib resistance capacities (Fig. 5d–f). Conversely, the inactivation of AKT signaling effectively abolished SOX9 upregulation caused by CD73 overexpression, together with abolished effects on sphere formation and Lenvatinib resistance capacities (Fig. 5g–i). These data imply that CD73 modulated SOX9 expression in an AKT-dependent manner.

We then purified CD73+ and CD73– cells, and treated CD73+ cells with MK-2206 and CD73– cells with Sc-79. We found that AKT inhibition reduced the expression of SOX9 in CD73+ cells, whereas AKT activation greatly increased SOX9 expression in CD73– cells (Fig. 5j). Further, as expected, AKT inhibition attenuated sphere formation and Lenvatinib resistance capacities in CD73+ cells, while AKT activation achieved the opposite effects in CD73– cells (Fig. 5k, l). Finally, to confirm the critical role of SOX9 in the regulatory process, we knocked down SOX9 expression in SC-79-treated CD73-low expression cells. This substantially attenuated the effects of AKT activation on CSC traits, as evidenced by decreased EpCAM expression, sphere formation capacity, and Lenvatinib resistance potentials (Fig. 5m–o). Hence, our data clearly demonstrated that CD73 modulates SOX9 expression by activating AKT signaling.

### CD73 activates SOX9 transcription through c-Myc

**Fig. 6:**
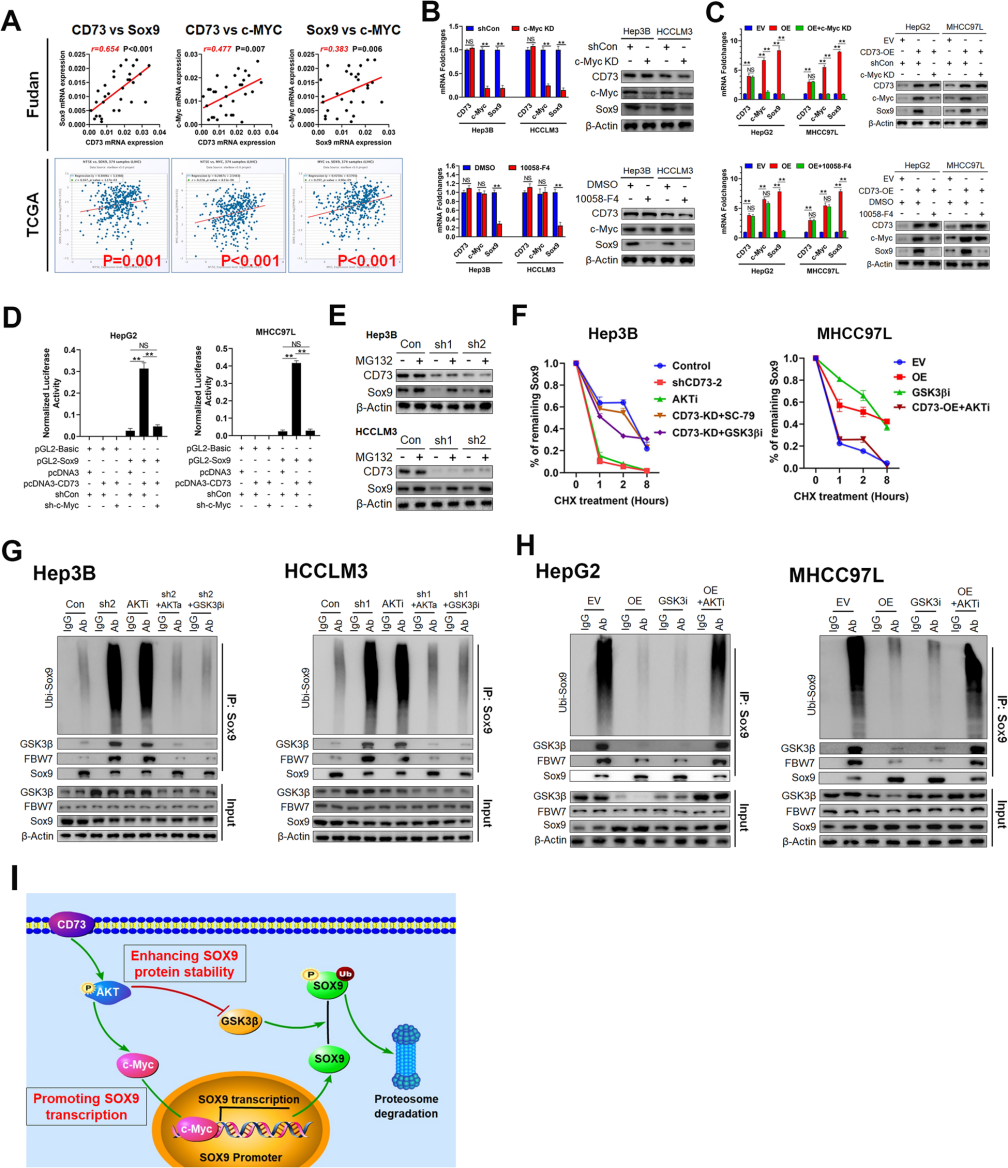
CD73 triggers SOX9 transcription by c-Myc and enhances Sox9 protein stability via inhibiting GSK3β activity. **a** Correlations between CD73, c-Myc, and SOX9 mRNA levels were analyzed based on RT-PCR results from our cohort (upper panel) and RNA-sequencing data from TCGA database (lower panel). **b** Effects of c-Myc knockdown (upper) or c-Myc antagonist treatment (lower) on SOX9 mRNA and protein expression levels in CD73-high HCC cells. **c** Effects of c-Myc knockdown (upper) or c-Myc antagonist treatment (lower) on SOX9 mRNA and protein expression levels in CD73-overexpressed HCC cells. **d** HepG2 (left) and MHCC97L (right) cells were co-transfected with indicated plasmids and the activities of SOX9 promoter were assessed by luciferase reporter assays. **e** Effects of proteasome degradation inhibitor, MG132, on protein expression level of SOX9 in CD73-knockdown Hep3B (upper) and HCCLM3 (lower) cells. **f** SOX9 protein stability in HCC cells received indicated treatments and exposed to a time-course treatment with CHX. **g** Ubiquitination assay of SOX9 in Hep3B (left) or HCCLM3 (right) cells received indicated treatments. Transfected cells were treated with MG132 for 6 h. **h** Ubiquitination assay of SOX9 in HepG2 (left) or MHCC97L (right) cells received indicated treatments. **i** Simplified diagram of the present study. Transfected cells were treated with MG132 for 6 h. Throughout the figure, “*” indicated *P* < 0.05, “**” indicated *P* < 0.01, and “***” indicated *P* < 0.001 by two-tailed *t* test or Mann–Whitney test

An increase in SOX9 expression might reflect activated SOX9 transcription and/or enhanced SOX9 protein stability. Indeed, SOX9 mRNA levels were positively correlated with CD73 expression (Fig. 3a). c-Myc serves as a key downstream target of AKT signaling, inducing the transcription of various genes upon AKT activation [36]. Importantly, the results from our cohort and the TCGA database showed that CD73 mRNA expression was positively correlated with both SOX9 and c-Myc, and that SOX9 expression was significantly correlated with that of c-Myc (Fig. 6a). Thus, we hypothesized that CD73 triggered SOX9 transcription via c-Myc.

In support of this, we first observed notable SOX9 downregulation at both the mRNA and protein level following the silencing of c-Myc in CD73-high expression cells (Fig. 6b upper). Moreover, the treatment of cells with the specific c-Myc inhibitor 10058-F4 significantly reduced SOX9 expression (Fig. 6b lower). Interestingly, we found that either c-Myc silencing or inhibitor treatment abolished the effect of CD73 overexpression on promoting SOX9 expression in CD73-low expression cells (Fig. 6c). Similarly, c-Myc silencing or inhibition significantly reduced SOX9 mRNA and protein levels in CD73+ fractions (Additional file 6: Figure S4A and B). Luciferase reporter assays demonstrated significantly higher SOX9 promoter activity when SOX9 and c-Myc were co-transfected into 293 T cells (Additional file 6: Figure S4C). Moreover, CD73 overexpression markedly activated the SOX9 promoter, while this effect was almost completely abolished by c-Myc silencing in CD73-low expression cells (Fig. 6d). Similar results were observed in sorted CD73– cells (Additional file 6: Figure S4D). Together, these data imply that CD73 mainly promotes SOX9 transcription through c-Myc.

### CD73 prevents SOX9 ubiquitination and proteasome degradation by inhibiting GSK3β

Active GSK3β phosphorylates SOX9, resulting in SOX9 degradation via the ubiquitin–proteasome pathway [34]. Because GSK3β activity was greatly hindered by CD73 through activating AKT [37], we next explored whether CD73 used this strategy to enhance SOX9 protein stability. We observed that the proteasome inhibitor MG132 partially restored SOX9 expression in CD73-silenced cells (Fig. 6e), suggesting that CD73 also contributes to the stability of SOX9 protein. Next, we detected a significant reduction in SOX9 protein stability when CD73 was silenced or AKT signaling was inhibited, as evidenced by a shorter SOX9 half-life. However, either an AKT agonist or GSK3β inhibitor effectively restored SOX9 stability caused by CD73 knockdown. By contrast, CD73 overexpression or the use of a GSK3β inhibitor greatly prolonged the SOX9 half-life in CD73-low expression cells, whereas an AKT inhibitor attenuated the effect of CD73 overexpression (Fig. 6f**,** Additional file 6: Figure S4E).

A previous study reported that residue T236 was critical for GSK3β-induced phosphorylation, resulting in SOX9 degradation [34]. To confirm this, we co-transfected wild-type SOX9 (WT-SOX9) or mutant SOX9 (SOX9-T236A) with GSK3β into 293 T cells. We found that GSK3β markedly promoted WT-SOX9 ubiquitination levels, resulting in a reduction in SOX9 expression. However, GSK3β failed to induce SOX9-T236A ubiquitination, also reducing SOX9 expression (Additional file 6: Figure S4F). Immunoprecipitation followed by western blotting was performed to determine SOX9 ubiquitination levels under different treatments. CD73 knockdown or AKT inhibition greatly enhanced the interaction between SOX9 and GSK3β/FBW7, resulting in increased SOX9 ubiquitination levels. However, AKT activation or GSK3β inhibition abolished the effects of CD73 knockdown on SOX9 ubiquitination (Fig. 6g). By contrast, CD73 overexpression and GSK3β inhibition markedly reduced the interaction between SOX9 and GSK3β/FBW7, leading to lower SOX9 ubiquitination levels, while AKT inhibition attenuated the effects of CD73 overexpression (Fig. 6h). Collectively, our data demonstrated that CD73 both promoted SOX9 transcription and enhanced SOX9 protein stability by activating AKT signaling (Fig. 6i).

### The combined analysis of CD73 and SOX9 is a promising approach to evaluate prognosis in HCC

**Fig. 7:**
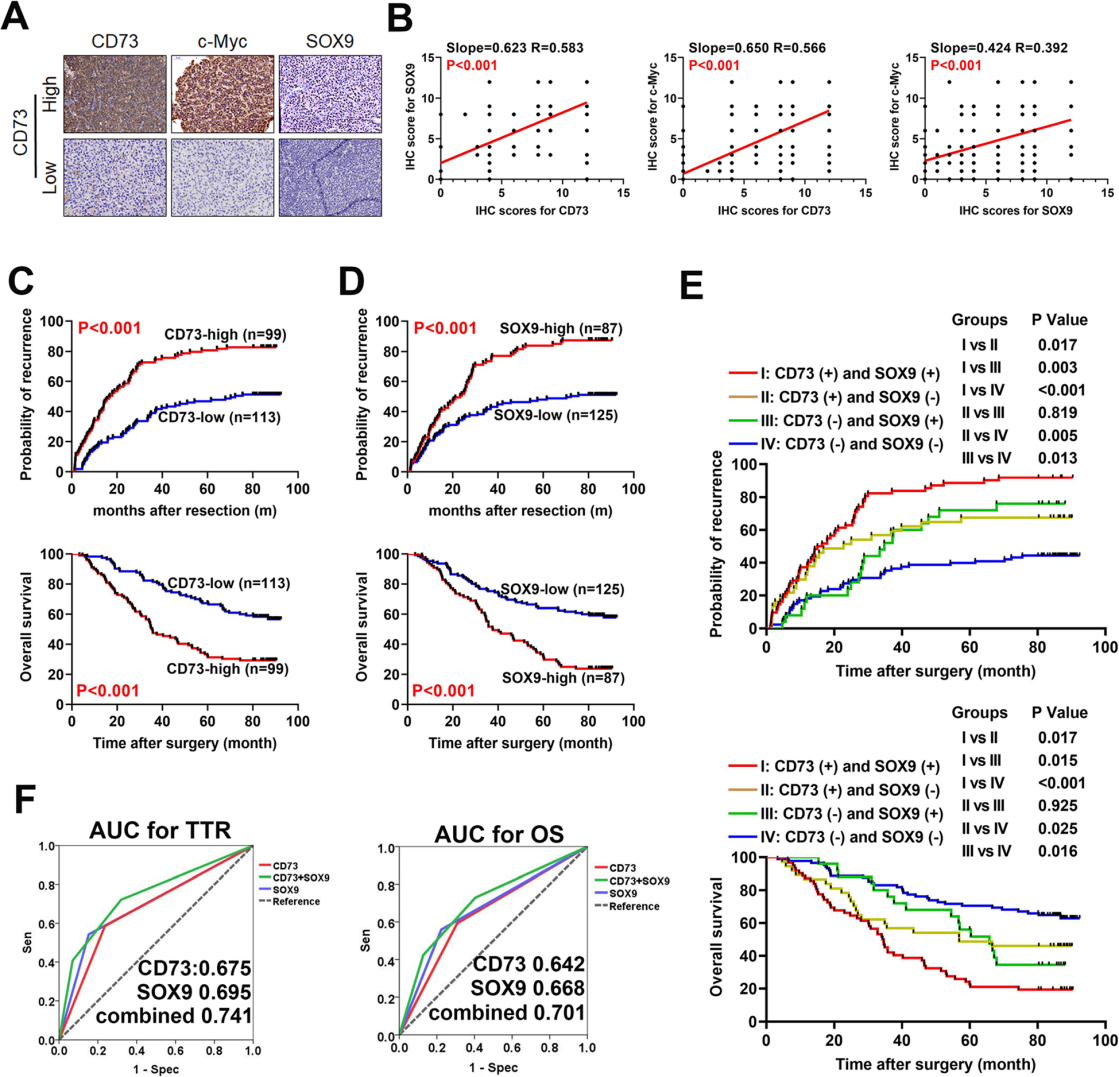
Combination of SOX9 and CD73 achieved satisfactory power on predicting prognosis in HCC. **a** Representative images of IHC staining for CD73, c-Myc, and SOX9. **b** Correlations between CD73, c-Myc, and SOX9 protein expressions. **c** Kaplan-Meier curve analysis of TTR (upper) and OS (lower) of HCC patients after curative resection according to CD73 expression level. **d** Kaplan-Meier curve analysis of TTR (upper) and OS (lower) of HCC patients after curative resection according to SOX9 expression level. **e** Prognostic significance of the combination of CD73 and SOX9 with respect to TTR (upper) and OS (lower) after curative resection. **f** Prognostic prediction performances of CD73, SOX9, or combination for TTR (left) and OS (right) were assessed by ROC curve analysis

We observed positive correlations between CD73 and SOX9 (*r* = 0.583, Fig. 7a, b), as well as between c-Myc and SOX9 and CD73 and c-Myc, which confirmed our earlier results based on mRNA expression. High expression of CD73 or SOX9 indicated a significantly worse HCC prognosis, as evidenced by shorter TTR and OS (all *P* < 0.001, Fig. 7c, d). Importantly, multivariate Cox regression identified both CD73 and SOX9 as independent predictors for shorter TTR [CD73: HR 2.251, 95% confidence interval (CI) 1.533–3.304, *P* < 0.001; SOX9: HR 1.697, 95% CI 1.138–2.478, *P* = 0.009] as well as OS [CD73: HR 1.981, 95% CI 1.208–2.998, *P* = 0.001; SOX9: HR 1.734, 95% CI 1.139–2.642, *P* = 0.010]. Therefore, we speculated that the combined analysis of CD73 and SOX9 would provide a more powerful tool to predict the prognosis of HCC patients. To validate this, we stratified HCC patients into four subgroups: (i) CD73-high and SOX9-high (*n* = 62); (ii) CD73-high and SOX9-low (*n* = 37); (iii) CD73-low and SOX9-high (*n* = 25); and (iv) CD73-low and SOX9-low (*n* = 88). TTR was significantly shorter in group i than groups ii (*P* = 0.017), iii (*P* = 0.003), and iv (*P* < 0.001). Patients in group ii (*P* = 0.005) or group iii (*P* = 0.013) also experienced a shorter TTR than those in group iv. However, TTR showed no significant difference between groups ii and iii (*P* = 0.819, Fig. 7e). Similarly, OS was significantly shorter in group i than groups ii (*P* = 0.017), iii (*P* = 0.015), and iv (P < 0.001). Patients in group ii (*P* = 0.025) or group iii (*P* = 0.016) experienced a shorter OS than those in group iv. However, OS did not differ significantly between groups ii and iii (*P* = 0.925, Fig. 7e). ROC curve analysis demonstrated that combining the analyses of CD73 and SOX9 exerted a greater power in distinguishing patients with worse prognosis (AUC-ROC; TTR: CD73 0.675, SOX9 0.695, combined 0.741; OS: CD73 0.642, SOX9 0.668, combined 0.701; Fig. 7f). Collectively, our data suggest that this combination is a promising tool for predicting prognosis in HCC patients.

## Discussion

Tumor heterogeneity is widely accepted, and the CSC model has been verified in various types of solid tumors including HCC [38]. Here, we report CD73 as a novel surface marker of CSC in HCC. We found that CD73 expression was essential for the capacities of self-renewal, differentiation, and the generation of new tumors. Importantly, we showed that the CD73+ cell fraction, but not CD73– cells, exerted typical CSC traits. Further investigations revealed that CD73 promoted CSC traits through dual AKT-dependent mechanisms: activating SOX9 transcription via c-Myc, and preventing SOX9 degradation by inhibiting GSK3β. Moreover, CD73 expression positively correlated with SOX9, suggesting that a combination of these two biomarkers has the potential to precisely predict the prognosis of HCC patients.

CD73 is a well-known surface marker for identifying mesenchymal stem cells [23]. Recent investigations revealed the potential for CD73+ photoreceptor precursors in future cell replacement therapy [39], and showed that CD73 could mark a multipotent stromal population [40]. CD73 was also reported to mark a fraction of mammary cells endowed with lineage plasticity [41]. These findings indicate the importance of CD73 in regulating stemness while increasing evidence suggests a role for CD73 in regulating CSC traits in several types of solid tumors [42, 43]. However, there was no specific data to connect CD73 with CSCs in HCC.

We previously revealed CD73 to be a critical oncogene for HCC progression that could trigger epithelial–mesenchymal transition [23], which is considered to confer stemness traits to cancer cells [2]. This strongly suggested that CD73 is a potential marker for identifying CSCs in HCC. Here, we found that CD73 was not only enriched in HCC spheres, but that it was also essential for the sphere formation capacity of HCC cells. Interestingly, we found CD73 also mainly depended on its enzyme activity to promote CSC traits in HCC (Additional file 7: Figure S5). To further determine whether CD73 was an ideal marker for liver CSC, we used MACS sorting and employed CD73 expression modulation to show that CD73 expression was essential for self-renewal, differentiation, and in vivo tumor propagation. Consistently, CD73+ HCC cells exhibited a significantly greater stemness potential than their CD73– counterparts. Notably, CD73 was also closely associated with an undifferentiated phenotype, as evidenced by the increased expression of stemness genes. Moreover, single-cell sorting showed that CD73+ HCC cells could differentiate into CD73– cells, but the reverse did not occur. Collectively, our data clearly show that CD73 is a novel, ideal, and promising surface marker for HCC.

Lenvatinib was recently approved as a novel molecular target regimen for first-line therapy in HCC [44]; this has resulted in a meaningful improvement in several second endpoints including disease-free progression [45]. However, resistance to Lenvatinib is observed in clinical practice [44], which greatly hinders the therapeutic effects and contributes to poor prognosis. Here, we found that overexpression of CD73 conferred HCC cells with significant resistance to Lenvatinib. Moreover, purified CD73+ cells exhibited outstanding drug resistance compared with their CD73– counterparts. Colony formation assays of the effects of CD73 on Lenvatinib resistance reflected the survival potential of HCC cells under Lenvatinib treatment. Thus, our data demonstrated that CD73 is a critical regulator contributing to Lenvatinib resistance; therefore, indicating that targeting of CD73 or elimination of CD73+ cells is a promising strategy for overcoming Lenvatinib resistance and prolonging OS. Intriguingly, CD73 is also critical for promoting other multiple kinase inhibitor resistance, such as sorafenib or Cabozantinib (Additional file 8: Figure S6), which suggests the potential role of CD73 in sustaining multiple kinase resistance of HCC, and more investigations are needed to confirm our findings.

Accumulating evidence has indicated that aberrant activation of AKT signaling is a vital process for maintaining CSC traits in HCC [11, 20, 46]. We previously found that CD73 effectively activated AKT signaling via the Rap1/P110β cascade [23]. Because CD73 appears to be a novel biomarker for CSCs in HCC, we speculated that CD73 also regulated CSC traits by activating AKT. In support of this, our data showed that AKT inactivation successfully hampered CSC traits in CD73-high expression cell lines or sorted CD73+ cells, whereas AKT activation triggered by SC-79 achieved the opposite effects in CD73-low expression cell lines or sorted CD73– cells. These results clearly confirmed our hypothesis and demonstrated the involvement of AKT signaling in CD73-regulated CSC traits.

SOX9 is an important transcription factor for regulating high mobility group box DNA binding and transactivation domains [34]. Previous studies implicated the contributions of SOX9 to stemness characteristics in HCC [47, 48]. However, the regulation of SOX9 transcription and protein stability was not fully investigated in HCC. Here, we showed that SOX9 is a critical downstream regulator of CD73 in HCC. Intriguingly, CD73 upregulated SOX9 expression via two distinct mechanisms: promoting SOX9 transcription by c-Myc and promoting SOX9 protein stability by inhibiting GSK3β activity, which resulted in an enhancement effect to further facilitate stemness characteristics. Although SOX9 was previously shown to activate Wnt signaling [49] which triggered Myc expression, the regulatory relationship between SOX9 and c-Myc remained vague in HCC. Here, we observed that c-Myc knockdown dramatically inhibited SOX9 mRNA expression, whereas SOX9 silencing had no significant effect on c-Myc expression, indicating the vital role of c-Myc in regulating SOX9 transcription in HCC. However, we failed to identify specific DNA-binding seeds of c-Myc on the SOX9 promoter (data not shown). Therefore, we speculate that c-Myc serves as a coregulator for other factors to indirectly enable SOX9 transcription. We plan to elucidate the mechanism underlying c-Myc-mediated SOX9 transcription in future work. It is conceivable that SOX9 is phosphorylated at the A236 residue by GSK3β, resulting in proteasomal degradation [34]. Because GSK3β is mainly inactivated by AKT [37], we further inferred that CD73 also enhances SOX9 protein stability via AKT activation. Here, we observed that CD73 could act as a “switch” to control SOX9 ubiquitination following proteasome degradation through inhibiting GSK3β by activating AKT signaling. Importantly, our findings also implicate a new regulatory mechanism of GSK3β in restricting CSC traits in HCC.

## Conclusions

In summary, our present study revealed CD73 as a novel surface marker for identifying CSC in HCC. CD73 sustained CSC traits both by upregulating SOX9 expression and maintaining its protein stability. Our findings suggest a potential target for overcoming Lenvatinib resistance and a more comprehensive understanding of the regulatory mechanism involved in liver CSC.

## Supplementary information

**Additional file 1:** Supplementary Tables; Description: Table S1 and S2.

**Additional file 2:** Supplementary figure legends.

**Additional file 3:** Figure S1 Description: CD73 expression was increased in HCC spheres.

**Additional file 4:** Figure S2 Description: Dynamic change pattern of CD73 according to serial differentiation assays.

**Additional file 5:** Figure S3 Description: CD73 positive percentages in indicated HCC cell lines.

**Additional file 6:** Figure S4 Description: CD73 triggers SOX9 transcription by c-Myc and enhances Sox9 protein stability via inhibiting GSK3β activity.

**Additional file 7:** Figure S5 Description: CD73 mainly depended on its enzyme activity to promote CSC traits in HCC.

**Additional file 8:** Figure S6 CD73 was critical for the resistance to sorafenib or Cabozantinib in HCC.

## Data Availability

The datasets used and/or analyzed during the current study are available from the corresponding author on reasonable request.

## Abbreviations

CHX: Cycloheximide
CSC: Cancer stem cell
GSK3β: Glycogen synthase kinase 3-beta
HCC: Hepatocellular carcinoma
IHC: Immunohistochemistry
IP: Immunoprecipitation
MACS: Magnetic bead cell sorting
NOD-SCID: Non-obese diabetic severe combined immunodeficiency
OS: Overall survival
TCGA: The cancer genome atlas
TTR: Time to recurrence
WB: Western blot

## References

[1] Yin Z, Dong C, Jiang K, Xu Z, Li R, Guo K (2019). Heterogeneity of cancer-associated fibroblasts and roles in the progression, prognosis, and therapy of hepatocellular carcinoma. J Hematol Oncol.

[2] Jayachandran A, Dhungel B, Steel JC (2016). Epithelial-to-mesenchymal plasticity of cancer stem cells: therapeutic targets in hepatocellular carcinoma. J Hematol Oncol.

[3] Lu C, Rong D, Zhang B, Zheng W, Wang X, Chen Z (2019). Current perspectives on the immunosuppressive tumor microenvironment in hepatocellular carcinoma: challenges and opportunities. Mol Cancer.

[4] Nault JC, Ningarhari M, Rebouissou S, Zucman-Rossi J (2019). The role of telomeres and telomerase in cirrhosis and liver cancer. Nat Rev Gastroenterol Hepatol..

[5] Calderaro J, Ziol M, Paradis V, Zucman-Rossi J (2019). Molecular and histological correlations in liver cancer. J Hepatol.

[6] Wu Q, Zhou L, Lv D, Zhu X, Tang H (2019). Exosome-mediated communication in the tumor microenvironment contributes to hepatocellular carcinoma development and progression. J Hematol Oncol.

[7] Kanwal F, Singal AG (2019). Surveillance for hepatocellular carcinoma: current best practice and future direction. Gastroenterology..

[8] Yin X, Zhang BH, Zheng SS, Gao DM, Qiu SJ, Wu WZ (2015). Coexpression of gene Oct4 and Nanog initiates stem cell characteristics in hepatocellular carcinoma and promotes epithelial-mesenchymal transition through activation of Stat3/snail signaling. J Hematol Oncol.

[9] Fan Z, Duan J, Wang L, Xiao S, Li L, Yan X (2019). PTK2 promotes cancer stem cell traits in hepatocellular carcinoma by activating Wnt/beta-catenin signaling. Cancer Lett.

[10] Cao J, Zhao M, Liu J, Zhang X, Pei Y, Wang J (2019). RACK1 promotes self-renewal and Chemoresistance of Cancer stem cells in human hepatocellular carcinoma through stabilizing Nanog. Theranostics..

[11] Gu Y, Wei X, Sun Y, Gao H, Zheng X, Wong LL (2019). miR-192-5p silencing by genetic aberrations is a key event in hepatocellular carcinomas with Cancer stem cell features. Cancer Res.

[12] Majumdar A, Curley SA, Wu X, Brown P, Hwang JP, Shetty K (2012). Hepatic stem cells and transforming growth factor beta in hepatocellular carcinoma. Nat Rev Gastroenterol Hepatol.

[13] Lv H, Lv G, Han Q, Yang W, Wang H (2018). Noncoding RNAs in liver cancer stem cells: the big impact of little things. Cancer Lett.

[14] Gordeeva O (2018). Cancer-testis antigens: unique cancer stem cell biomarkers and targets for cancer therapy. Semin Cancer Biol.

[15] Ji J, Wang XW (2012). Clinical implications of cancer stem cell biology in hepatocellular carcinoma. Semin Oncol.

[16] Huo X, Han S, Wu G, Latchoumanin O, Zhou G, Hebbard L (2017). Dysregulated long noncoding RNAs (lncRNAs) in hepatocellular carcinoma: implications for tumorigenesis, disease progression, and liver cancer stem cells. Mol Cancer.

[17] Klingenberg M, Matsuda A, Diederichs S, Patel T (2017). Non-coding RNA in hepatocellular carcinoma: mechanisms, biomarkers and therapeutic targets. J Hepatol.

[18] Bharti R, Dey G, Mandal M (2016). Cancer development, chemoresistance, epithelial to mesenchymal transition and stem cells: a snapshot of IL-6 mediated involvement. Cancer Lett.

[19] Chen HA, Kuo TC, Tseng CF, Ma JT, Yang ST, Yen CJ (2016). Angiopoietin-like protein 1 antagonizes MET receptor activity to repress sorafenib resistance and cancer stemness in hepatocellular carcinoma. Hepatology..

[20] Wei X, You X, Zhang J, Zhou C (2019). MicroRNA-1305 inhibits the Stemness of LCSCs and tumorigenesis by repressing the UBE2T-dependent Akt-signaling pathway. Mol Ther Nucleic Acids.

[21] Huan HB, Yang DP, Wen XD, Chen XJ, Zhang L, Wu LL (2017). HOXB7 accelerates the malignant progression of hepatocellular carcinoma by promoting stemness and epithelial-mesenchymal transition. J Exp Clin Cancer Res.

[22] Zhang PP, Wang PQ, Qiao CP, Zhang Q, Zhang JP, Chen F (2016). Differentiation therapy of hepatocellular carcinoma by inhibiting the activity of AKT/GSK-3beta/beta-catenin axis and TGF-beta induced EMT with sophocarpine. Cancer Lett.

[23] Ma XL, Shen MN, Hu B, Wang BL, Yang WJ, Lv LH (2019). CD73 promotes hepatocellular carcinoma progression and metastasis via activating PI3K/AKT signaling by inducing Rap1-mediated membrane localization of P110beta and predicts poor prognosis. J Hematol Oncol.

[24] Ma XL, Jiang M, Zhao Y, Wang BL, Shen MN, Zhou Y (2018). Application of serum Annexin A3 in diagnosis, outcome prediction and therapeutic response evaluation for patients with hepatocellular carcinoma. Ann Surg Oncol.

[25] Ma XL, Zhou JY, Gao XH, Tian L, Wu J, Zhang CY (2016). Application of the albumin-bilirubin grade for predicting prognosis after curative resection of patients with early-stage hepatocellular carcinoma. Clin Chim Acta.

[26] Zhou SL, Yin D, Hu ZQ, Luo CB, Zhou ZJ, Xin HY, et al. A Positive Feedback Loop Between Cancer Stem-Like Cells and Tumor-Associated Neutrophils Controls Hepatocellular Carcinoma Progression. Hepatology. 2019. 10.1002/hep.30630 Epub ahead of print. 10.1002/hep.30630 30933361

[27] Ma XL, Sun YF, Wang BL, Shen MN, Zhou Y, Chen JW (2019). Sphere-forming culture enriches liver cancer stem cells and reveals Stearoyl-CoA desaturase 1 as a potential therapeutic target. BMC Cancer.

[28] Fu PY, Hu B, Ma XL, Yang ZF, Yu MC, Sun HX (2019). New insight into BIRC3: a novel prognostic indicator and a potential therapeutic target for liver cancer. J Cell Biochem.

[29] Ho DW, Tsui YM, Sze KM, Chan LK, Cheung TT, Lee E (2019). Single-cell transcriptomics reveals the landscape of intra-tumoral heterogeneity and stemness-related subpopulations in liver cancer. Cancer Lett.

[30] Wu J, Zhu P, Lu T, Du Y, Wang Y, He L (2019). The long non-coding RNA LncHDAC2 drives the self-renewal of liver cancer stem cells via activation of hedgehog signaling. J Hepatol.

[31] Liu Y, Zhang JB, Qin Y, Wang W, Wei L, Teng Y (2013). PROX1 promotes hepatocellular carcinoma metastasis by way of up-regulating hypoxia-inducible factor 1alpha expression and protein stability. Hepatology..

[32] Li JH, Liu S, Zhou H, Qu LH, Yang JH (2014). starBase v2.0: decoding miRNA-ceRNA, miRNA-ncRNA and protein-RNA interaction networks from large-scale CLIP-Seq data. Nucleic Acids Res.

[33] Zhang L, Chen J, Ning D, Liu Q, Wang C, Zhang Z (2019). FBXO22 promotes the development of hepatocellular carcinoma by regulating the ubiquitination and degradation of p21. J Exp Clin Cancer Res.

[34] Suryo RA, Savov V, Brunner A, Bolin S, Weishaupt H, Malyukova A (2016). FBW7 suppression leads to SOX9 stabilization and increased malignancy in medulloblastoma. EMBO J.

[35] Yu Q, Li W, Xie D, Zheng X, Huang T, Xue P (2018). PI3Kgamma promotes vascular smooth muscle cell phenotypic modulation and transplant arteriosclerosis via a SOX9-dependent mechanism. EBioMedicine..

[36] Zhang F, Li K, Yao X, Wang H, Li W, Wu J (2019). A miR-567-PIK3AP1-PI3K/AKT-c-Myc feedback loop regulates tumour growth and chemoresistance in gastric cancer. EBioMedicine..

[37] Chen S, Cao W, Yue P, Hao C, Khuri FR, Sun SY (2011). Celecoxib promotes c-FLIP degradation through Akt-independent inhibition of GSK3. Cancer Res.

[38] Chen C, Zhao S, Karnad A, Freeman JW (2018). The biology and role of CD44 in cancer progression: therapeutic implications. J Hematol Oncol.

[39] Gagliardi G, Ben MK, Chaffiol A, Slembrouck-Brec A, Conart JB, Nanteau C (2018). Characterization and transplantation of CD73-positive photoreceptors isolated from human iPSC-derived retinal Organoids. Stem Cell Reports.

[40] Breitbach M, Kimura K, Luis TC, Fuegemann CJ, Woll PS, Hesse M (2018). In vivo labeling by CD73 Marks multipotent stromal cells and highlights endothelial heterogeneity in the bone marrow niche. Cell Stem Cell.

[41] Samanta D, Park Y, Ni X, Li H, Zahnow CA, Gabrielson E (2018). Chemotherapy induces enrichment of CD47(+)/CD73(+)/PDL1(+) immune evasive triple-negative breast cancer cells. Proc Natl Acad Sci U S A.

[42] Lupia M, Angiolini F, Bertalot G, Freddi S, Sachsenmeier KF, Chisci E (2018). CD73 regulates Stemness and epithelial-Mesenchymal transition in ovarian Cancer-initiating cells. Stem Cell Reports..

[43] Katsuta E, Tanaka S, Mogushi K, Shimada S, Akiyama Y, Aihara A (2016). CD73 as a therapeutic target for pancreatic neuroendocrine tumor stem cells. Int J Oncol.

[44] Al-Salama ZT, Syed YY, Scott LJ (2019). Lenvatinib: a review in hepatocellular carcinoma. Drugs..

[45] Kudo M, Finn RS, Qin S, Han KH, Ikeda K, Piscaglia F (2018). Lenvatinib versus sorafenib in first-line treatment of patients with unresectable hepatocellular carcinoma: a randomised phase 3 non-inferiority trial. Lancet..

[46] Jang JW, Song Y, Kim SH, Kim JS, Kim KM, Choi EK (2017). CD133 confers cancer stem-like cell properties by stabilizing EGFR-AKT signaling in hepatocellular carcinoma. Cancer Lett.

[47] Xiao Y, Sun Y, Liu G, Zhao J, Gao Y, Yeh S (2019). Androgen receptor (AR)/miR-520f-3p/SOX9 signaling is involved in altering hepatocellular carcinoma (HCC) cell sensitivity to the Sorafenib therapy under hypoxia via increasing cancer stem cells phenotype. Cancer Lett.

[48] Kawai T, Yasuchika K, Ishii T, Miyauchi Y, Kojima H, Yamaoka R (2016). SOX9 is a novel cancer stem cell marker surrogated by osteopontin in human hepatocellular carcinoma. Sci Rep.

[49] Santos JC, Carrasco-Garcia E, Garcia-Puga M, Aldaz P, Montes M, Fernandez-Reyes M (2016). SOX9 elevation acts with canonical WNT signaling to drive gastric Cancer progression. Cancer Res.

