# Supplementary material for: CD73 sustained cancer-stem-cell traits by promoting SOX9 expression and stability in hepatocellular carcinoma
Source: J Hematol Oncol. 2020 Feb 5;13:11. doi: 10.1186/s13045-020-0845-z (PMC7003355; doi:10.1186/s13045-020-0845-z)
Supplement: Supplementary file 1 — Additional file 1: Supplementary Tables; Description: Table S1 and S2. [file 13045_2020_845_MOESM1_ESM.docx]

| **Table S1. Primers used in present study** | | |
| --- | --- | --- |
| **Gene name** | **Forward 5’-3’** | **Reverse 5’-3’** |
| CD73 | GATCGAGCCACTCCTCAAA | GCCCATCAGAAGTGAC |
| EpCAM | GAAGGCTGAGATAAAGGAGATGGG | TTAACGATGGAGTCCAAGTTCTGG |
| Nanog | ATGCCTGTGATTTGTGGGCC | GCCAGTTGTTTTTCTGCCAC |
| SOX2 | TACAGCATGTCCTACTCGCAG | GAGGAAGAGGTAACCACAGGG |
| OCT4 | AGGGCTTCTCCTTCTGGGTCT | TGAGAAAGGAGACCCAGCAG |
| SOX9 | CGAAATCAACGAGAAACTGGAC | ATTTAGCACACTGATCACACG |
| c-Myc | TACCCTCTCAACGACAGCAG | TCTTGACATTCTCCTCGGTG |
| Albumin | TGCAACTCTTCGTGAAACCTATG | ACATCAACCTCTGGTCTCACC |
| CK8 | ATCAGCTCCTCGAGCTTC | TCCAGGAACCGTACCTTGTC |
| β-Actin | TTGTTACAGGAAGTCCCTTGCC | ATGCTATCACCTCCCCTGTGTG |

| **Table 2. Information of antibodies** | | | |
| --- | --- | --- | --- |
| **Target protein** | **Manufacturer** | **Application** | **Dilution** |
| CD73 | Abcam (ab54217) | WB | 1:1000 |
| EpCAM | Abcam (ab71916) | WB | 1:1000 |
| SOX2 | Abcam (ab79351) | WB | 1:1000 |
| Oct4 | Abcam (ab109183) | WB | 1:1000 |
| SOX9 | Abcam (ab185966) | WB | 1:1000 |
|  | Abcam (ab185966) | IHC | 1:200 |
|  | Abcam (ab185230) | IP | 1:50 |
| c-Myc | Abcam (ab32072) | WB | 1:800 |
|  | Abcam (ab32072) | IHC | 1:100 |
| Albumin | Abcam (ab137885) | WB | 1:1000 |
| CK8 | Abcam (ab53280) | WB | 1:750 |
| AKT | CST (#4685) | WB | 1:1000 |
| pAKT | CST (#4060) | WB | 1:1000 |
| GSK3β | CST (#9832) | WB | 1:1000 |
| pGSK3β | CST (#9323) | WB | 1:1000 |
| Ubiquitin | Abcam (ab7780) | WB | 1:1000 |
| β-Actin | CST (#4970) | WB | 1:2000 |
