# Supplementary material for: CD73 sustained cancer-stem-cell traits by promoting SOX9 expression and stability in hepatocellular carcinoma
Source: J Hematol Oncol. 2020 Feb 5;13:11. doi: 10.1186/s13045-020-0845-z (PMC7003355; doi:10.1186/s13045-020-0845-z)
Supplement: Supplementary file 2 — Additional file 2: Supplementary figure legends. [file 13045_2020_845_MOESM2_ESM.docx]

**Supplementary Figure Legends**

**Figure S1. CD73 expression was increased in HCC spheres.** (A) CD73 expression levels of sphere cells derived from clinical fresh HCC samples and corresponding paired parental cells were determined by WB assays. (B) CD73 expression levels of sphere cells derived from HCC cell lines and corresponding paired parental cells were determined by WB assays.

**Figure S2. Dynamic change pattern of CD73 according to serial differentiation assays.** (A) Expression levels of CD73 and EpCAM among the 1^st^, 2^nd^, 3^rd^ spheres and corresponding differentiated sphere cells in Hp3B (Left) and HCCLM3 (right) cells. (B) Expression levels of CD73 and EpCAM among the 1^st^, 2^nd^, 3^rd^ spheres and corresponding differentiated sphere cells in two sphere cells derived from fresh clinical HCC samples.

**Figure S3. CD73 positive percentages in indicated HCC cell lines.**

**Figure S4. CD73 triggers SOX9 transcription by c-Myc and enhances Sox9 protein stability via inhibiting GSK3β activity.** (A) Effects of c-Myc knockdown on SOX9 mRNA and protein expression levels in sorted CD73+ Hep3b (left two panels) and HCCLM3 (right two panels). (B) Effects of c-Myc antagonist treatment on SOX9 mRNA and protein expression levels in sorted CD73+ Hep3b (left two panels) and HCCLM3 (right two panels). (C) HEK293T cells were co-transfected with indicated plasmids and the activities of SOX9 promoter were assessed by luciferase reporter assays. (D) CD73- Hep3B (left) and HCCLM3 (right) cells were co-transfected with indicated plasmids and the activities of SOX9 promoter were assessed by luciferase reporter assays. (E) Western blot analysis of CD73 and SOX9 protein stability in HCC cells received indicated treatments and exposed to a time-course treatment with CHX. (F) Ubiquitination assay of wild-type SOX9 and mutant SOX9 (T236A) in HEK293T cells co-transfected with GSK3β and ubiquitin. Transfected cells were treated with MG132 for 6 hours.

**Figure S5: Description: CD73 mainly depended on its enzyme activity to promote CSC traits in HCC.**

**Figure S6: CD73 was critical for the resistance to sorafenib or Cabozantinib in HCC.** (A) CD73 knockdown significantly sensitized Hep3B cells towards Cabozantinib treatment. (B) CD73 knockdown significantly sensitized HCCLM3 cells towards Cabozantinib treatment. (C) CD73 knockdown significantly sensitized Hep3B cells towards sorafenib treatment. (D) CD73 knockdown significantly sensitized HCCLM3 cells towards sorafenib treatment.
