# Supplementary figures and images for: CD73 sustained cancer-stem-cell traits by promoting SOX9 expression and stability in hepatocellular carcinoma
Source: J Hematol Oncol. 2020 Feb 5;13:11. doi: 10.1186/s13045-020-0845-z (PMC7003355; doi:10.1186/s13045-020-0845-z)

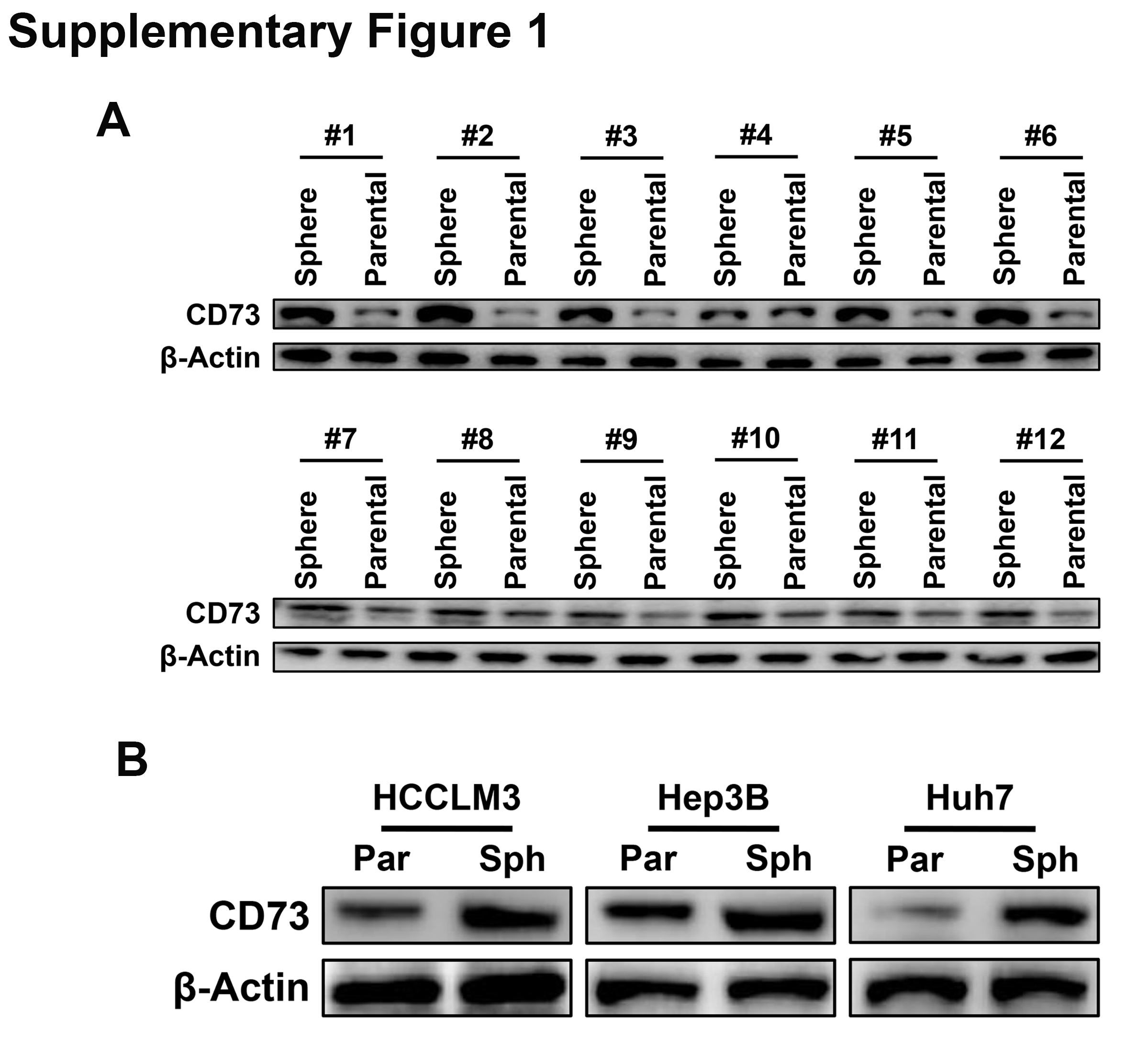

Supplement: Supplementary file 3 — Additional file 3: Figure S1 Description: CD73 expression was increased in HCC spheres. [file 13045_2020_845_MOESM3_ESM.tif]

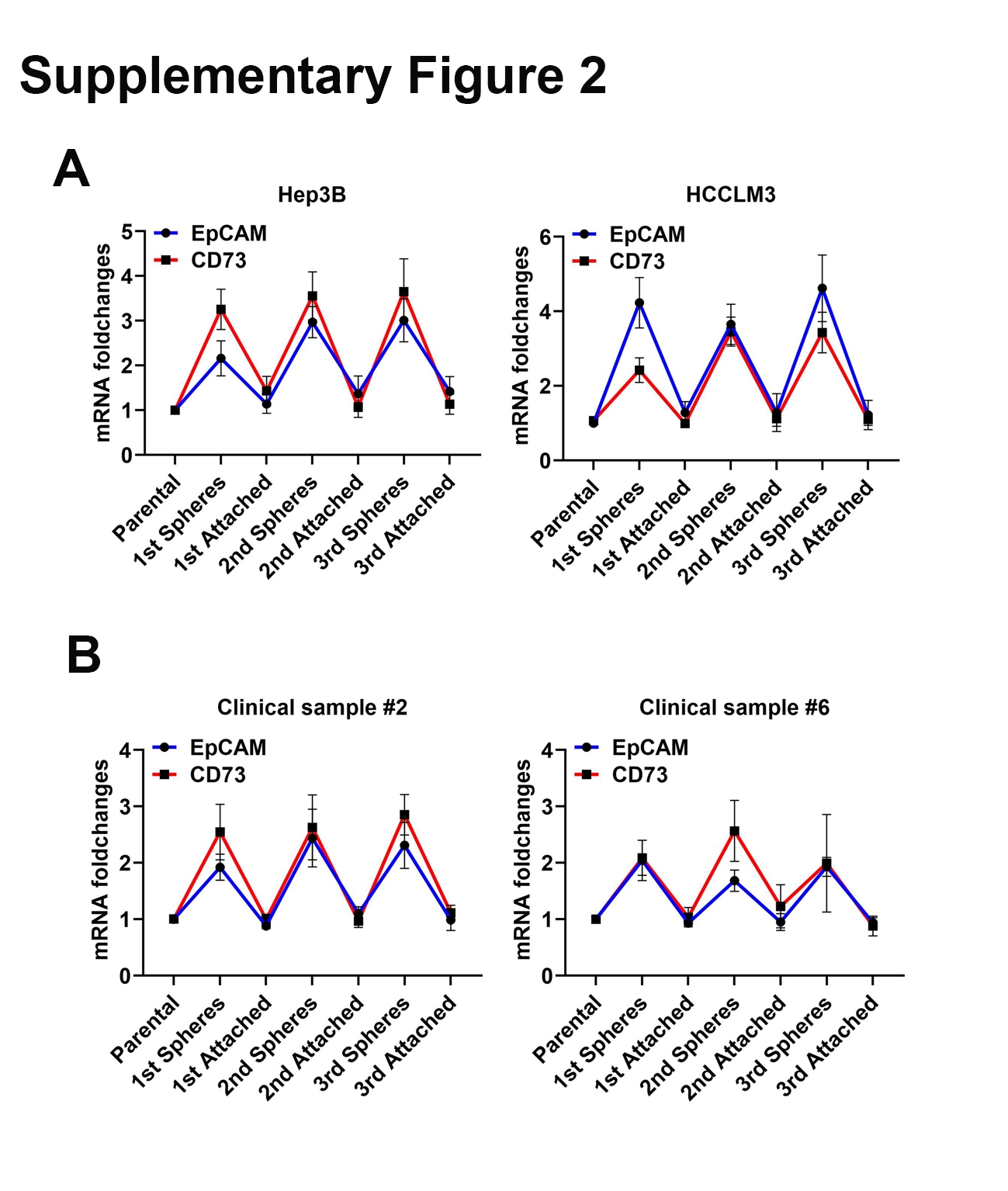

Supplement: Supplementary file 4 — Additional file 4: Figure S2 Description: Dynamic change pattern of CD73 according to serial differentiation assays. [file 13045_2020_845_MOESM4_ESM.tif]

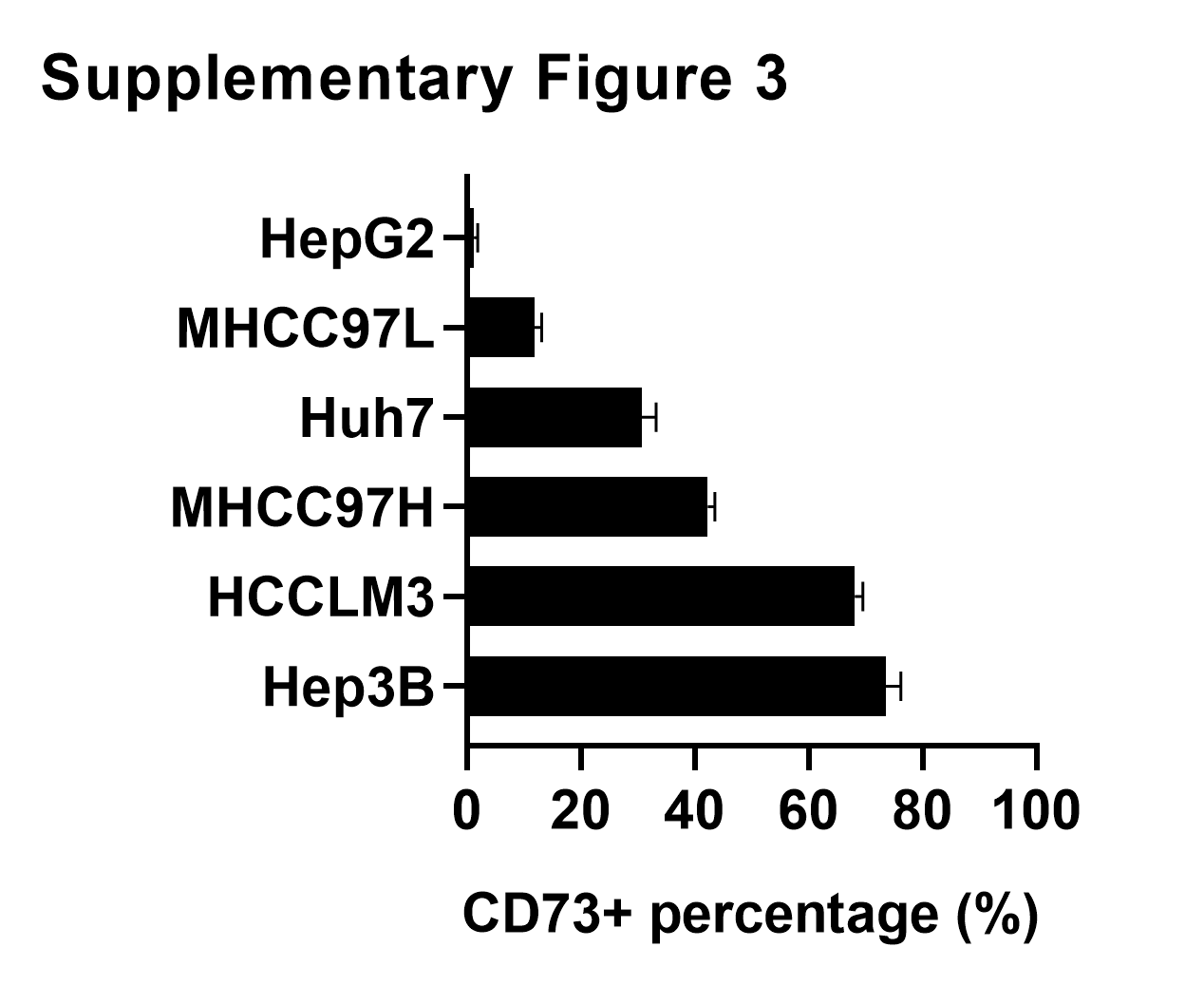

Supplement: Supplementary file 5 — Additional file 5: Figure S3 Description: CD73 positive percentages in indicated HCC cell lines. [file 13045_2020_845_MOESM5_ESM.tif]

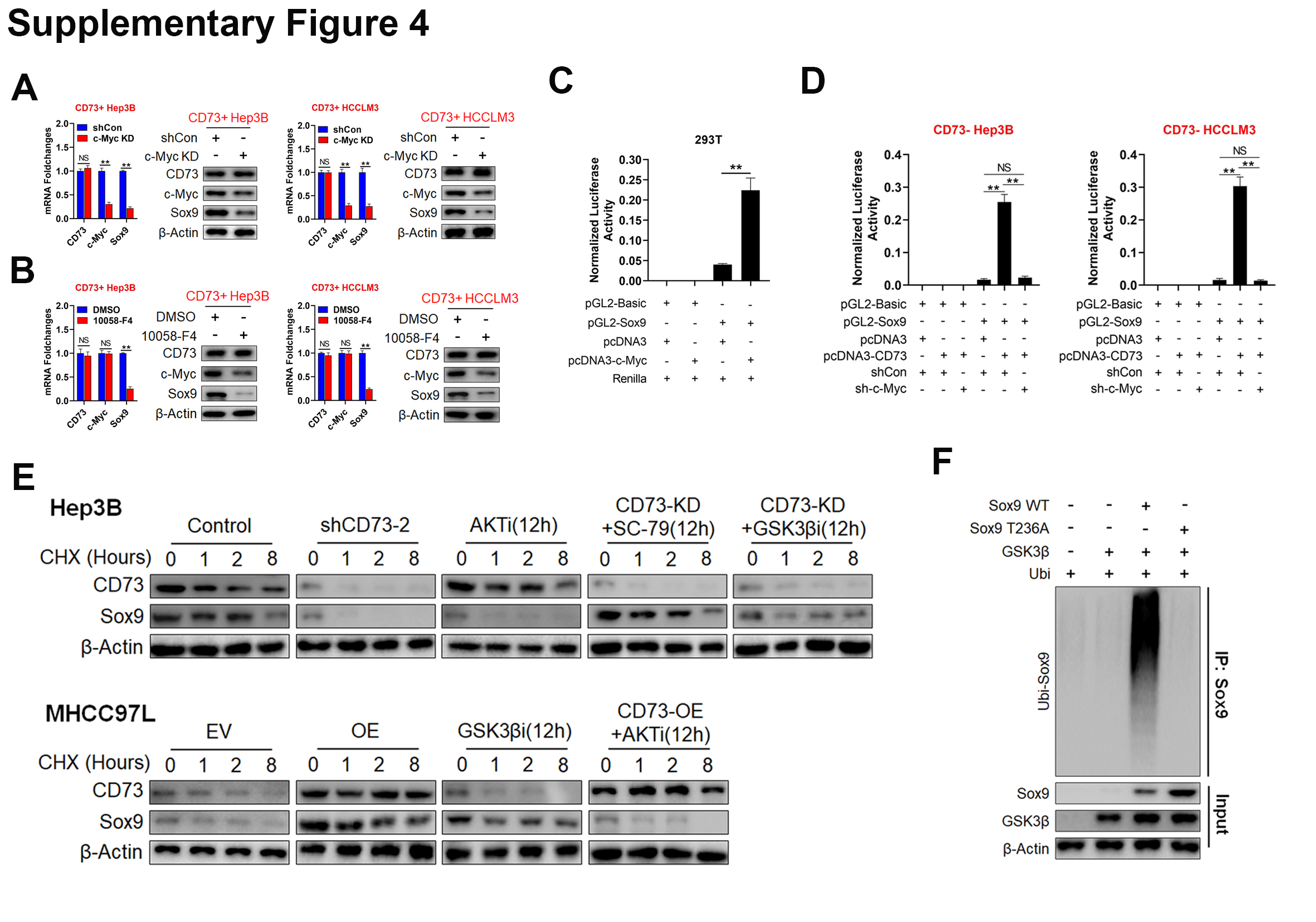

Supplement: Supplementary file 6 — Additional file 6: Figure S4 Description: CD73 triggers SOX9 transcription by c-Myc and enhances Sox9 protein stability via inhibiting GSK3β activity. [file 13045_2020_845_MOESM6_ESM.tif]

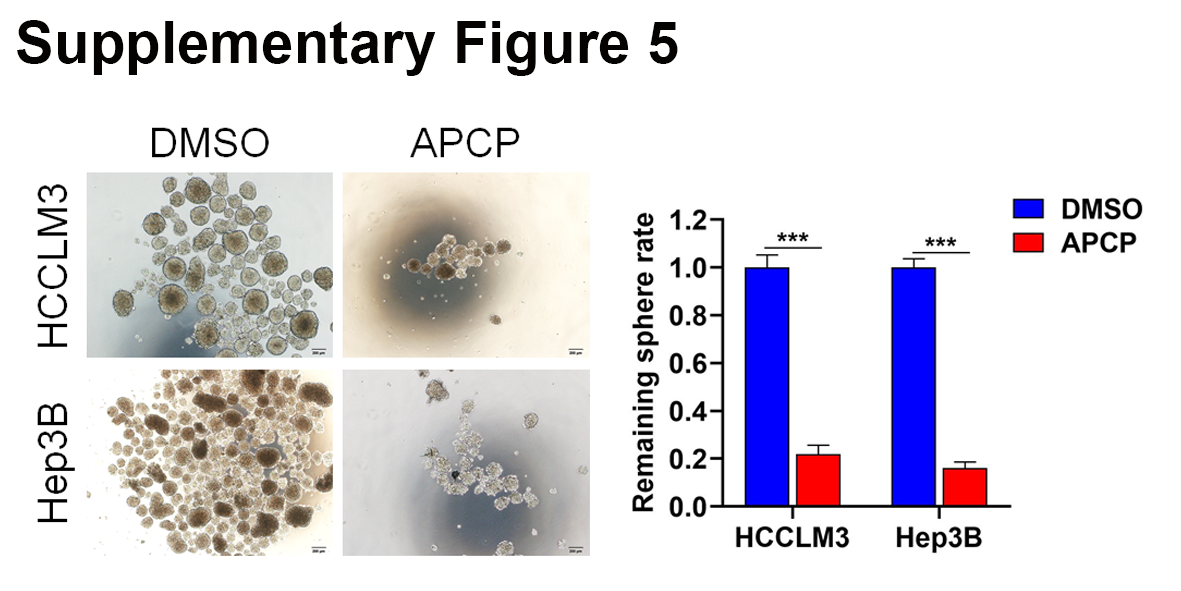

Supplement: Supplementary file 7 — Additional file 7: Figure S5 Description: CD73 mainly depended on its enzyme activity to promote CSC traits in HCC. [file 13045_2020_845_MOESM7_ESM.tif]

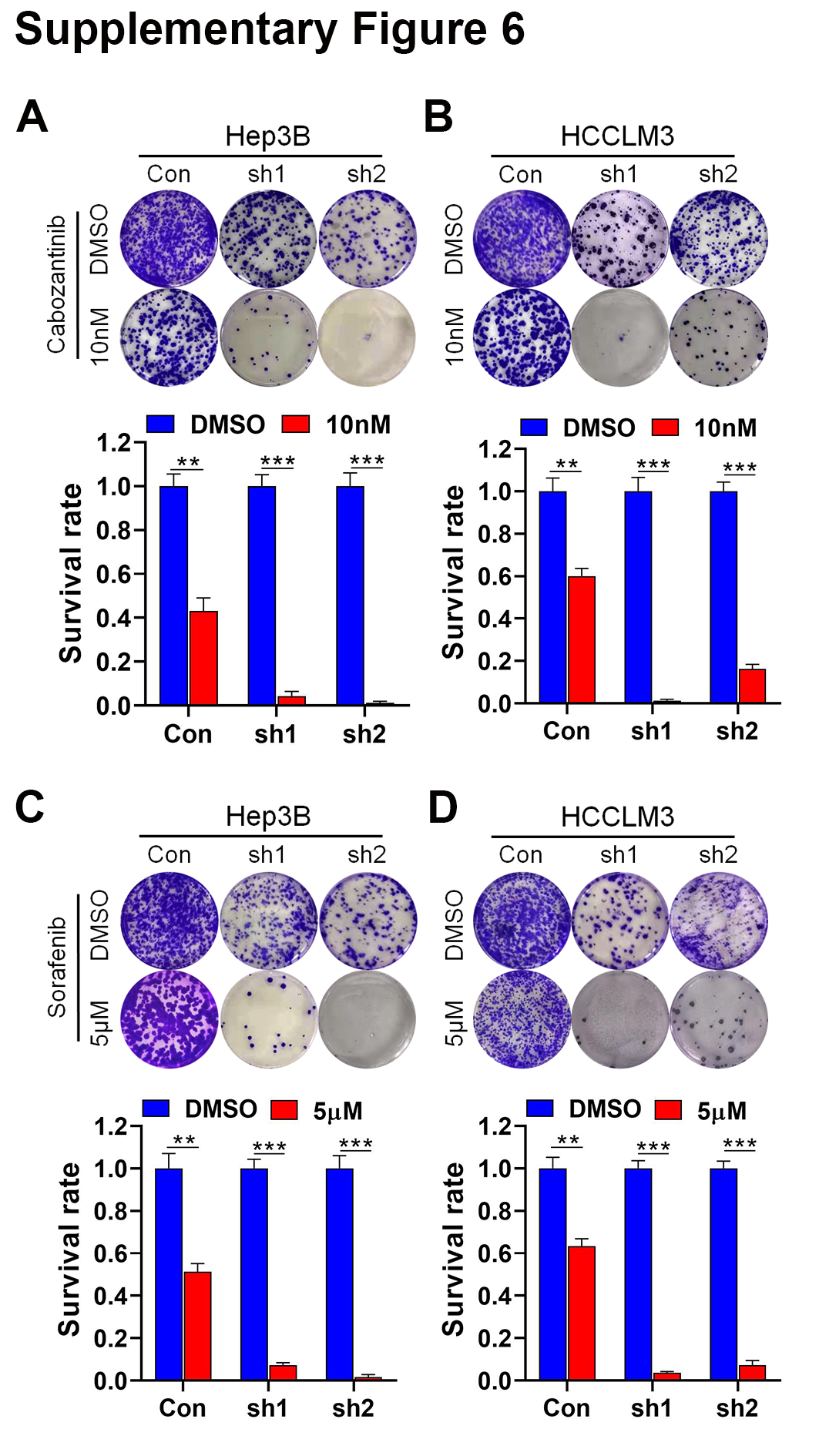

Supplement: Supplementary file 8 — Additional file 8: Figure S6 CD73 was critical for the resistance to sorafenib or Cabozantinib in HCC. [file 13045_2020_845_MOESM8_ESM.tif]
